# Real-time CBCT imaging and motion tracking via a single arbitrarily-angled x-ray projection by a joint dynamic reconstruction and motion estimation (DREME) framework

**DOI:** 10.1088/1361-6560/ada519

**Published:** 2025-01-21

**Authors:** Hua-Chieh Shao, Tielige Mengke, Tinsu Pan, You Zhang

**Affiliations:** 1 The Medical Artificial Intelligence and Automation (MAIA) Laboratory, Department of Radiation Oncology, University of Texas Southwestern Medical Center, Dallas, TX 75390, United States of America; 2 Department of Imaging Physics, University of Texas MD Anderson Cancer Center, Houston, TX 77030, United States of America

**Keywords:** real-time imaging, motion estimation, dynamic CBCT reconstruction, motion model, x-ray, deep learning

## Abstract

*Objective.* Real-time cone-beam computed tomography (CBCT) provides instantaneous visualization of patient anatomy for image guidance, motion tracking, and online treatment adaptation in radiotherapy. While many real-time imaging and motion tracking methods leveraged patient-specific prior information to alleviate under-sampling challenges and meet the temporal constraint (<500 ms), the prior information can be outdated and introduce biases, thus compromising the imaging and motion tracking accuracy. To address this challenge, we developed a framework dynamic reconstruction and motion estimation (DREME) for real-time CBCT imaging and motion estimation, without relying on patient-specific prior knowledge. *Approach.* DREME incorporates a deep learning-based real-time CBCT imaging and motion estimation method into a dynamic CBCT reconstruction framework. The reconstruction framework reconstructs a dynamic sequence of CBCTs in a data-driven manner from a standard pre-treatment scan, without requiring patient-specific prior knowledge. Meanwhile, a convolutional neural network-based motion encoder is jointly trained during the reconstruction to learn motion-related features relevant for real-time motion estimation, based on a single arbitrarily-angled x-ray projection. DREME was tested on digital phantom simulations and real patient studies. *Main Results.* DREME accurately solved 3D respiration-induced anatomical motion in real time (∼1.5 ms inference time for each x-ray projection). For the digital phantom studies, it achieved an average lung tumor center-of-mass localization error of 1.2 ± 0.9 mm (Mean ± SD). For the patient studies, it achieved a real-time tumor localization accuracy of 1.6 ± 1.6 mm in the projection domain. *Significance.* DREME achieves CBCT and volumetric motion estimation in real time from a single x-ray projection at arbitrary angles, paving the way for future clinical applications in intra-fractional motion management. In addition, it can be used for dose tracking and treatment assessment, when combined with real-time dose calculation.

## 1. Introduction

Radiotherapy aims to maximize local tumor control while minimizing radiotoxicity to the healthy tissues/organs adjacent to target tumors (Verellen *et al* 2007). To accomplish such goals, sophisticated treatment planning and dose delivery techniques, such as volumetric modulated arc therapy (Rashid *et al* 2021) and stereotactic body radiotherapy (Kimura *et al* 2022), were developed to yield dose distributions highly conformal to target tumors (Bernier *et al* 2004). However, respiration-induced anatomical motion, particularly for tumors in the thoracic and abdominal regions, introduces variations in target shapes and locations (Shirato *et al* 2004), compromising the precision of radiotherapy. To address the motion-related uncertainties, patient-specific motion characteristics (e.g. motion patterns and amplitudes) are often quantified during treatment simulation and planning, based on which customized motion management strategies are applied to patients during the treatment (Keall *et al* 2006). Most motion management tools (e.g. respiratory gating, deep inspiration breath hold, or tumor tracking) require real-time imaging/motion signals to monitor daily patient motion when delivering each treatment (Keall *et al* 2019). However, until today, most of the monitoring signals are limited to surrogate or two-dimensional (2D) signals, including surface optical markers, interstitial fiducial markers, or x-ray fluoroscopy (Cui *et al* 2007, Xu *et al* 2014, Poulsen *et al* 2015, Sakata *et al* 2020). These 1D or 2D signals cannot not accurately reflect the 3D deformable motion (Roman *et al* 2012), or capture complicated nonlinear motion trajectories of organs and tumors (Langen and Jones 2001, Seppenwoolde *et al* 2002, Shirato *et al* 2004, White *et al* 2013). Therefore, 3D volumetric imaging is highly desired to capture instantaneous patient anatomy to achieve the most accurate tumor localization, allow intra-treatment dose tracking, and enable potential real-time treatment adaptation.

Currently, a major limitation of x-ray-based volumetric imaging is its temporal resolution. For respiratory motion (typically 3–5 s per cycle), the AAPM task group 264 recommended real-time tumor tracking or plan adaptation to have a temporal resolution of $ \unicode{x2A7D} $500 ms (Keall *et al* 2021). Within such a timeframe, very few 2D x-ray projections can be acquired. Reconstructing volumetric cone-beam computed tomography (CBCT) images from these projections is unfeasible by conventional reconstruction algorithms due to extreme under-sampling. Recently, the interest is growing in using deep leaning (DL)-based approaches to solve this problem (Mylonas *et al* 2021, Liu *et al* 2024). With usually-low inference latency, DL solutions are seen particularly suited for the real-time imaging problem. One type of DL approaches (Type I) attempts direct 3D image reconstruction from sparsely sampled 2D x-ray projections (Shen *et al* 2019, Ying *et al* 2019, Tong *et al* 2020, Shen *et al* 2022a, Zhou *et al* 2022, Zhang *et al* 2024, Zhu *et al* 2024). In particular, Shen *et al* (2019) developed a patient-specific volumetric reconstruction method from a single x-ray projection. Their network used a representation module to extract image features from a 2D x-ray projection, and then converting the 2D feature maps by a transformation module and a generation module to 3D feature maps and synthesizing the 3D image. Ying *et al* (2019) proposed to reconstruct a CT volume from two orthogonal x-ray projections, using generative adversarial learning. Their network comprised two 2D encoders and a 3D decoder that were linked by skip connections to bridge the 2D and 3D feature maps and to fuse the features extracted from the two orthogonal projections. However, reconstructing a 3D volume from a single or few 2D projections is a highly ill-posed inverse problem, thus the DL models can suffer from instability and generalizability issues. Moreover, the aforementioned networks were trained on x-ray projections at fixed gantry angles, rendering them impractical for radiotherapy treatments with rotating gantries.

The second type of DL works (Type II) utilizes deformable registration for real-time image/motion estimation and target localization (Wei *et al* 2020, Nakao *et al* 2022, Shao *et al* 2023). The registration-based approaches introduce prior knowledge (e.g. known patient-specific image and/or motion model), and infer 3D motion fields from limited on-board signals (e.g. one x-ray projection) to deform the patient-specific reference image to generate new images. The prior knowledge supplies additional (patient-specific) information to condition the ill-posed problem, and allows target tumors to be directly localized after registration through propagations of prior segmentations (thus removing the need of additional segmentations). In particular, Wei *et al* (2020) proposed a patient-specific convolutional neural network (CNN) to solve 3D motion fields from a single x-ray projection at arbitrary gantry angles. The angle-agnostic imaging was achieved by using an angle-dependent lung mask to extracted motion-related image features, followed by angle-dependent fully connected layers to map 2D image features to 3D motion fields. However, training fully connected layers separately for each projection angle is less practical and highly resource demanding. Recently, Shao *et al* (2023) developed a graph neural network-based, angle-agnostic DL model to estimate 3D deformable motion from an arbitrarily-angled x-ray projection. Instead of using angle-specific model layers, they incorporated the x-ray cone-beam projection geometry into the feature extraction process to allow patient-specific, angle-agnostic inference. However, although the registration-driven real-time imaging solutions hold great promises, they similarly face generalizability and robustness challenges. Generally, DL models, either type I or type II, require a large training dataset to properly learn and generalize to unseen scenarios. Due to the ill-condition of real-time imaging, most of such models are developed as patient-specific models to more effectively capture the patient traits. For patient-specific models, to overcome the size limits of patient-specific data, they usually rely on prior motion models to augment the training samples (e.g. Shen *et al* 2019, Wei *et al* 2020, Shao *et al* 2023). Typically, these motion models were derived from 4D-CTs acquired during treatment planning, and the 4D-CTs were augmented with the motion model to yield more random motion states. The motion model extracted from a planning 4D-CT, however, may not reflect varying breathing motion patterns observed during different treatments (Vergalasova and Cai 2020). The 4D-CT motion model also only captures breathing-related intra-scan motion, while other day-to-day anatomical motion cannot be modeled. The deformation-driven augmentations cannot model non-deformation-related anatomical variations, either (Zhang *et al* 2017). In addition, 4D-CT is commonly acquired through retrospective phase sorting, under the assumption of regular and periodic motion. However, patient motion can be highly irregular, leading to residual 4D-CT motion artifacts that further degrade the accuracy of its motion model (Yasue *et al* 2022).

**Figure 1.**
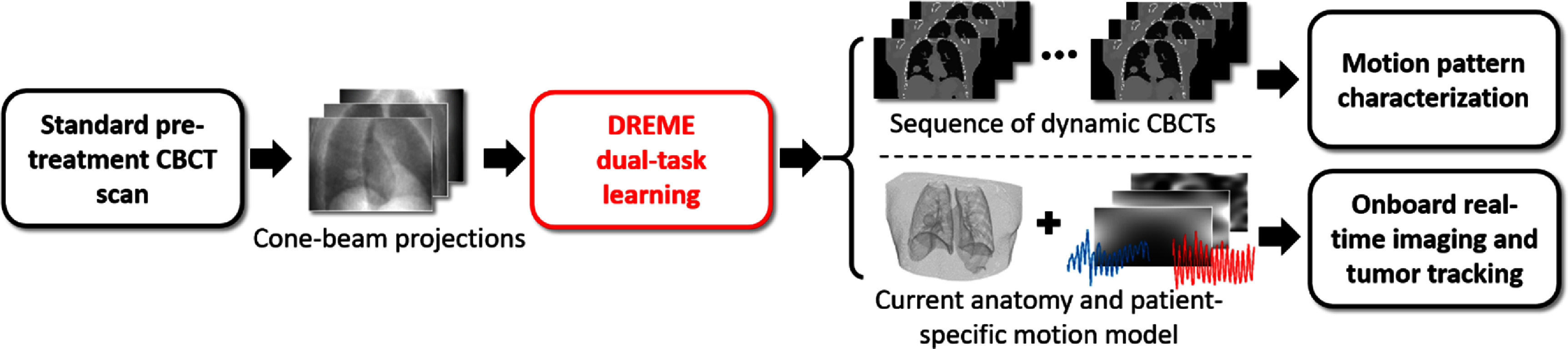
Workflow for dynamic CBCT reconstruction and real-time motion estimation. DREME uses cone-beam projections from a standard pre-treatment CBCT scan as input to simultaneously reconstruct a sequence of dynamic CBCTs (*upper route*), and a patient reference anatomy and its corresponding motion model (*lower route*) which are subsequently used for the real-time CBCT imaging and target tracking during treatment.

To overcome the limitations and challenges of previous methods, in this study we proposed a DL-based joint framework, dynamic reconstruction and motion estimation (DREME), for real-time CBCT imaging and tumor localization. DREME integrates a DL-based motion estimation module into a previously-developed dynamic CBCT reconstruction workflow (Shao *et al* 2024), solving two learning-based tasks in a single training session: 1. Reconstructing a pre-treatment, dynamic CBCT sequence to capture daily anatomy and motion patterns; and 2. Deriving a CNN motion encoder to allow real-time CBCT and motion inference from an arbitrarily-angled, intra-treatment x-ray projection. As shown in figure 1, the first learning task reconstructs a dynamic sequence of CBCT volumes (i.e. one CBCT volume for each x-ray projection) from a standard pre-treatment cone-beam scan to extract the most up-to-date patient anatomical and motion information without relying on patient-specific prior knowledge (Shao *et al* 2024). In contrast to the conventional motion-sorted 4D-CT/CBCT (e.g. Ou *et al* 2024, Wang *et al* 2024), dynamic CBCT offers a richer motion representation with much higher temporal resolution (∼15 frames/second), enabling the capture of irregular motion patterns and providing a more accurate characterization of motion dynamics. The second learning task uses the motion model directly learned from the pre-treatment dynamic CBCT reconstruction to derive a light-weight CNN motion encoder for real-time motion estimation, which can be used to deform a reference CBCT solved in dynamic reconstruction to real-time CBCT images. Compared with the previous DL-based real-time imaging methods, the dual task learning-based DREME framework includes both reconstruction and registration components, allowing it to combine the benefits from both worlds. Compared with the type I DL-based reconstruction methods, DREME deforms the real-time CBCT from a reconstruction-based pre-treatment CBCT scan, by solving motion from real-time singular x-ray projections. The pre-treatment CBCT brings in the most relevant prior information (immediate images of the same treatment session) to strongly condition the real-time imaging problem and stabilize its solution, without introducing generalizability issues. Under such conditioning, we are able to develop an angle-agnostic model, which has been challenging for previous type I methods. Compared with the type II DL-based registration methods, DREME uses newly reconstructed, pre-treatment dynamic CBCTs rather than planning 4D-CT images for motion modeling. The motion model learned from daily pre-treatment scans is not susceptible to day-to-day motion pattern variations. As the motion model is built on the pre-treatment dynamic CBCT images, any day-to-day anatomical variations, either deformation- or non-deformation-related, are naturally absorbed into the pre-treatment CBCTs and require no additional modeling. In addition, compared with 4D-CT, the dynamic CBCT of DREME is reconstructed to resolve both regular and irregular motion (Zhang *et al* 2023, Shao *et al* 2024), thus their images and the corresponding motion models are less susceptible to artifacts caused by irregular motion.

To the best of our knowledge, DREME is the first dual-task learning framework capable of real-time CBCT imaging and motion estimation without relying on patient-specific prior knowledge for model training. It makes a ‘one-shot’ learning technique, which only requires a single pre-treatment CBCT scan for training and does not need additional projections/images/motion models, rendering it highly data-efficient. We evaluated the DREME framework using a digital phantom simulation study and a multi-institutional lung patient dataset. Ablation studies were also performed on DREME to assess the contribution of its components, along with comparisons with other real-time imaging solutions.

## 2. Materials and methods

### 2.1. Dual-task learning DREME framework

#### 2.1.1. Overview of the dynamic CBCT reconstruction algorithm and the motion model

DREME achieves real-time CBCT imaging and markerless tumor tracking by combining dynamic CBCT reconstruction and real-time motion estimation into a single framework, without requiring any patient-specific prior information (figure 1). The reconstruction task (learning task 1) solves the latest patient anatomy and a patient-specific motion model immediately before each treatment, which are used by the real-time imaging task (learning task 2) for motion tracking and real-time CBCT rendering. The framework was adapted from our previous work on dynamic CBCT reconstruction called PMF-STINR (Shao *et al* 2024), with new components incorporated to allow simultaneous image reconstruction and motion encoder learning.

**Figure 2.**
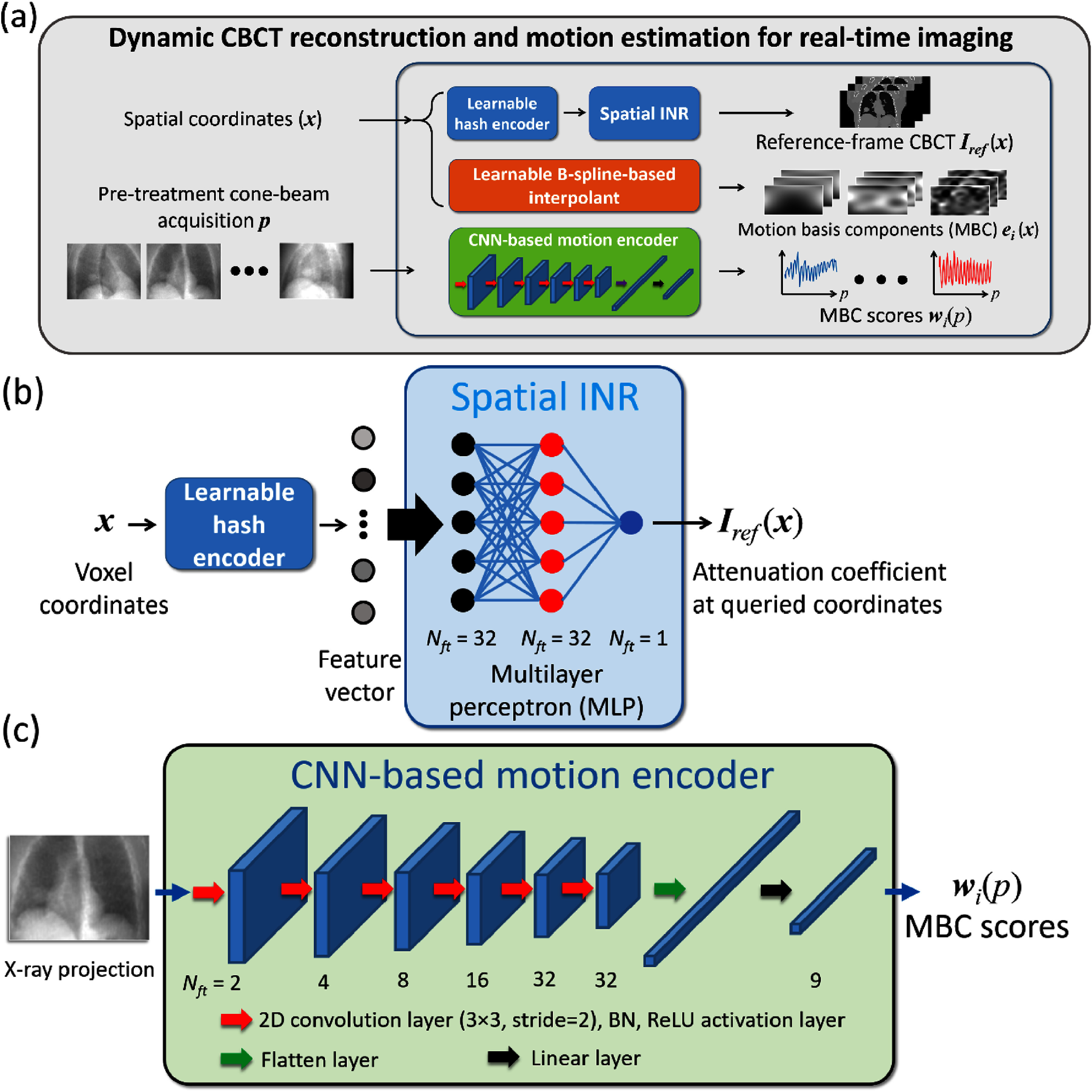
DREME network architecture. (a) DREME consists of a learnable hash encoder, a spatial implicit neural representation (INR), a learnable B-spline interpolant, and a convolutional neural network (CNN)-based motion encoder. It adopts a motion-compensated CBCT reconstruction approach based on a pre-treatment cone-beam acquisition ${\boldsymbol{p}}$, by joint deformable registration and reconstruction. The hash encoder and INR estimate the reference CBCT ${{\boldsymbol{I}}_{{\text{ref}}}}\left( {\boldsymbol{x}} \right)$, while the CNN-based motion encoder and the learnable B-spline interpolant estimate the projection-dependent (temporal) and spatial components of the deformation vector fields ${\boldsymbol{d}}\left( {{\boldsymbol{x}},{\text{ }}p} \right)$ (DVFs) with respect to the reference CBCT. Dynamic CBCTs ***I***(***x***, *p*) are derived by deforming the reference CBCT using the solved DVFs corresponding to each projection *p* of the cone-beam acquisition ${\boldsymbol{p}}$. (b) The spatial INR uses a three-layer multilayer perceptron (MLP) with periodic activation functions to represent the mapping from the voxel coordinate ***x*** to the attenuation coefficient ${{\boldsymbol{I}}_{{\text{ref}}}}\left( {\boldsymbol{x}} \right)$. *N*_ft_ denotes the feature number in each layer. (c) The CNN-based motion encoder takes an x-ray projection *p* as an input, extracting motion-related image features to estimate the corresponding MBC scores (i.e., temporal coefficients) ${{\boldsymbol{w}}_i}\left( p \right)$.

In general, DREME reconstructs a dynamic sequence of 3D CBCT volumes from a pre-treatment cone-beam projection set $\boldsymbol{p}$ (figure 1). For each CBCT scan, the anatomy captured at different time points is highly correlated. Therefore, the DREME algorithm took a joint deformable registration and reconstruction approach, assuming that the dynamic anatomy can be represented by a static reference anatomy ${{\boldsymbol{I}}_{{\text{ref}}}}\left( {\boldsymbol{x}} \right)$ and a time-varying, projection-dependent deformable vector field (DVF) ${\boldsymbol{d}}\left( {{\boldsymbol{x}},{\text{ }}p} \right)$ with respect to the reference anatomy, i.e,

\begin{equation*}{\boldsymbol{I}}\left( {{\boldsymbol{x}},{\text{ }}p} \right) = {\text{ }}{{\boldsymbol{I}}_{{\text{ref}}}}\left( {{\boldsymbol{x}} + {\boldsymbol{d}}\left( {{\boldsymbol{x}},{\text{ }}p} \right)} \right),\end{equation*}

where ***x*** denotes the voxel coordinates, and $p \in {\boldsymbol{p}}$. DREME represents the reference anatomy ${{\boldsymbol{I}}_{{\text{ref}}}}\left( {\boldsymbol{x}} \right)$ by implicit neural representation (INR) (Mildenhall *et al* 2022, Tewari *et al* 2022, Molaei *et al* 2023), by which the underlying mapping ${\boldsymbol{x}} \mapsto {{\boldsymbol{I}}_{{\text{ref}}}}\left( x \right)$ from a voxel coordinate ***x*** to the corresponding attenuation coefficient ${{\boldsymbol{I}}_{{\text{ref}}}}\left( {\boldsymbol{x}} \right)$ is implicitly parametrized by the learnable parameters of a neural network (figure 2(b)). Since reconstructing a dynamic sequence of 3D volumes can involve solving more than 10^8^ unknowns, dimension reduction is used to condition the ill-posed spatiotemporal inverse problem and avoid sub-optimal solutions. Particularly, we decomposed each DVF into low-rank spatiotemporal components by a data-driven motion model learned on the fly:

\begin{equation*}{\boldsymbol{d}}\left( {{\boldsymbol{x}},{\text{ }}p} \right) = {\text{ }}\mathop \sum \limits_{i = 1}^3 {{\boldsymbol{w}}_i}\left( p \right) \times {{\boldsymbol{e}}_i}\left( {\boldsymbol{x}} \right),\end{equation*}

where ${{\boldsymbol{w}}_i}\left( p \right)$ is represented by a CNN-based motion encoder (figure 2(c)), while the spatial components ${{\boldsymbol{e}}_i}\left( {\boldsymbol{x}} \right)$ is represented by a B-spline-based interpolant (figure 2(a)). We used three levels (*i*= 1, 2, 3) for the spatial decomposition, which has been demonstrated sufficient for representing respiratory motion (Li *et al* 2011). The control points of the B-spline interpolants are learnable parameters (Tegunov and Cramer 2019), and a hierarchical multi-resolution strategy (Shao *et al* 2024) was used so that each level *i* represents a different spatial scale. The spatial components ${{\boldsymbol{e}}_i}\left( {\boldsymbol{x}} \right)$ serve a basis set spanning a Hilbert sub-space of the solution space, thus called motion basis components (MBCs) in this work.

In the following subsections, we begin by summarizing the DREME network architecture, detailing the design and roles of the INR and CNN-based motion encoder in the DREME framework. Next, we present the learning objectives associated with learning tasks 1 and 2.

#### 2.1.2. DREME network architecture

Figure 2(a) illustrates the DREME network architecture, which comprises a spatial INR (figure 2(b)), a learnable B-spline-based interpolant, and a CNN-based motion encoder (figure 2(c)). As aforementioned, the spatial INR reconstructs the reference CBCT ${{\boldsymbol{I}}_{{\text{ref}}}}\left( {\boldsymbol{x}} \right)$ representing the reference anatomical template, and the B-spline interpolant and the CNN motion encoder respectively model the spatial ${{\boldsymbol{e}}_i}\left( {\boldsymbol{x}} \right)$ and the projection-dependent ${{\boldsymbol{w}}_i}\left( p \right)$ components in the low-rank motion model (equation (2)).

The spatial INR (figure 2(b)) continuously maps the input spatial space ${\boldsymbol{x}} \in {\mathbb{R}^3}$ to the corresponding image domain, using a three-layer multilayer perceptron (MLP) as a non-parametric universal function approximator. INRs have demonstrated high learning efficiency (Mildenhall *et al* 2022) and superior performance for representing complex functions, and have been applied to various medical image reconstruction and registration problems (Reed *et al* 2021, Shen *et al* 2022b, Zha *et al* 2022, Lin *et al* 2023, Zhang *et al* 2023). Before feeding the spatial coordinate ***x*** into the MLP, learnable hashing encoders (Muller *et al* 2022) are employed to enable DREME to capture high-frequency details of the underlying functions. The hash encoder utilizes a multi-resolution encoding scheme with learnable hash tables at each resolution level, mapping the input coordinates to a higher-dimensional feature vector to facilitate high-frequency learning. To acquire the whole anatomy, all voxel coordinates of the CBCT volume were sequentially inputted into the hash encoder and spatial INR, followed by gridding operations to a 3D volume. We used three-layer MLP with feature numbers of 32, 32, and 1 for the input, hidden, and output layers, respectively. A periodic activation function (SIREN (Sitzmann *et al* 2020)) was used for the hidden layer to further improve high-frequency learning. No activation function was used for the output layer.

The spatial components of the motion model (equation (2) are parametrized by a learnable B-spline-based interpolant, which provides a sparse and continuous representation of MBCs ${{\boldsymbol{e}}_i}\left( {\boldsymbol{x}} \right)$. The interpolant takes a voxel coordinate ***x*** as the input and computes the corresponding MBCs through B-spline interpolation, where the control points are learnable parameters (Tegunov and Cramer 2019). As described earlier, three spatial levels were used for $\left\{ {{{\boldsymbol{e}}_i}\left( {\boldsymbol{x}} \right)} \right\}_{i = 1}^3$. The lowest level used 6 × 6 × 6 grid of control points for each Cartesian direction (*x, y, z*), and the number of control points was doubled at each higher level to represent finer motion.

In contrast to our previous PMF-STINR model designed solely for the task of dynamic reconstruction (Shao *et al* 2024), DREME replaced PMF-STINR’s MLP-based motion encoder with a CNN-based motion encoder that directly infers the motion coefficients ${{\boldsymbol{w}}_i}\left( p \right)$ from a single x-ray projection (figure 2(c)), thus enabling the capability of real-time imaging. The motion encoder is trained to decipher the pre-treatment CBCT projections to represent the dynamic motion. In addition, it also serves to map future, intra-treatment x-ray projections into motion coefficients to solve instantaneous motion for real-time CBCT estimation. The motion encoder consists of six layers of 2D convolution layers with 3 × 3 convolution kernels. The feature maps of the layers comprise 2, 4, 8, 16, 32, and 32 channels, respectively. Each convolution layer is followed by a batch normalization layer and a rectified linear unit function (ReLU). After the last ReLU, the feature maps are flattened and processed by a linear layer with nine outputs. Each output channel represents an MBC score ${w_{i,k}}\left( p \right)$, where *i*= 1, 2, 3 and *k* = *x, y, z*. The subscripts *i* and *k* represent the index of the MBC levels (1, 2, and 3) and the index of the Cartesian components (*x, y*, and *z*), respectively.

##### 2.1.3. Learning task 1: dynamic CBCT reconstruction

**Figure 3.**
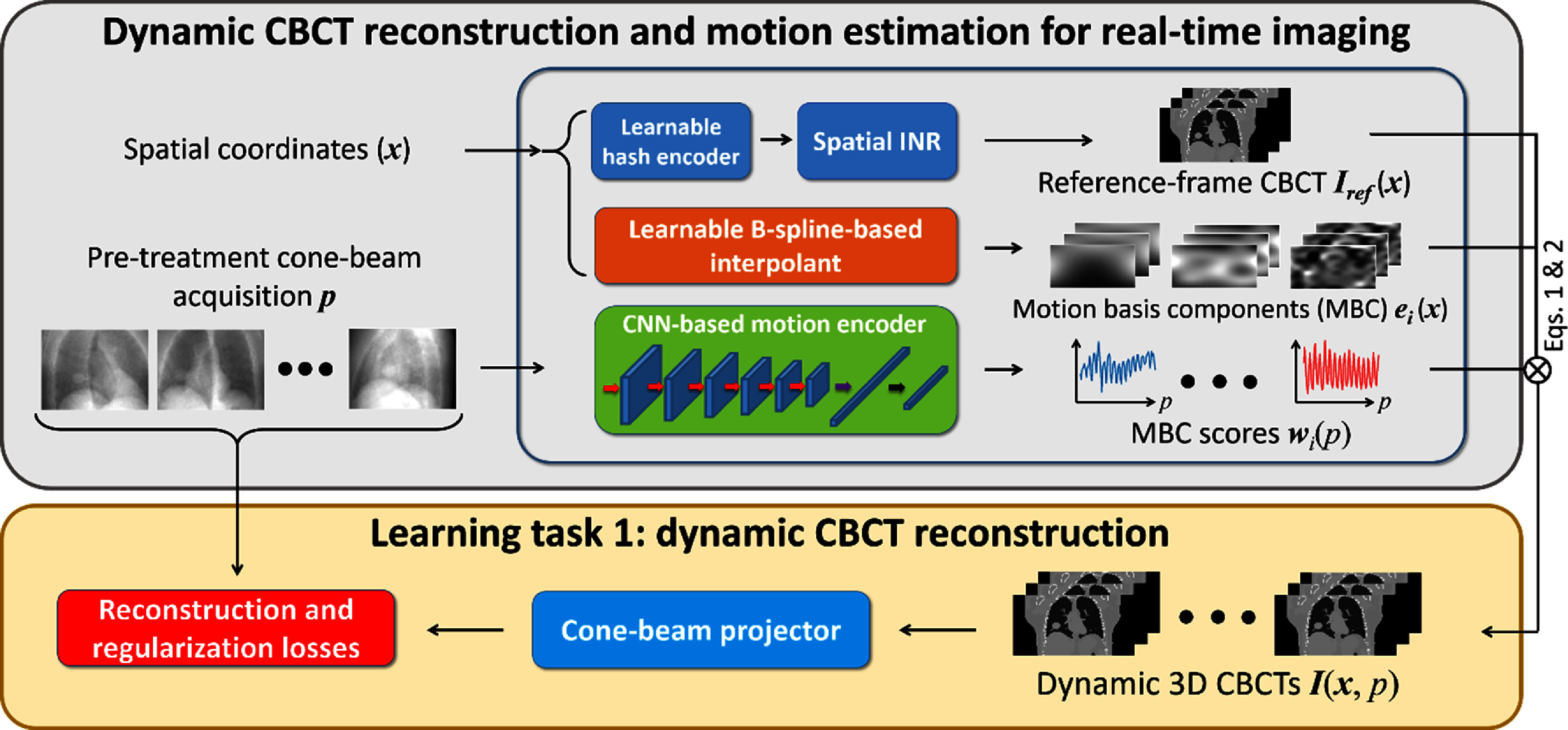
Learning task 1: dynamic CBCT reconstruction. The learning task of dynamic CBCT reconstruction is driven by maximizing the similarity between the digitally reconstructed radiographs (DRRs) of the motion-resolved CBCTs and the corresponding cone-beam projections. To regularize the ill-posed spatiotemporal inverse problem, regularization losses are incorporated into the training objectives.

In this subsection we present the learning objectives of the first learning task (figure 3). Two similarity loss functions were used to drive the training process of learning task 1. They were defined on the image- and projection-domain, respectively. The image-domain loss served as the training label to warm start the spatial INR, and the projection-domain loss drove the joint training process of the reference CBCT and the data-driven motion model. Specifically, the image-domain loss function $L_{{\text{sim}}}^{{\text{im}}}$ was defined as the mean squared error between the spatial INR ${{\boldsymbol{I}}_{{\text{ref}}}}\left( {\boldsymbol{x}} \right)$ and an approximate CBCT ${{\boldsymbol{I}}_{{\text{app}}}}\left( {\boldsymbol{x}} \right)$:

\begin{equation*}L_{{\text{sim}}}^{{\text{im}}} = \frac{1}{{{N_{{\text{voxel}}}}}}\mathop \sum \limits_{l = 1}^{{N_{{\text{voxel}}}}} {\left| {{{\boldsymbol{I}}_{{\text{ref}}}}\left( {{{\boldsymbol{x}}_l}} \right) - {{\boldsymbol{I}}_{{\text{app}}}}\left( {{{\boldsymbol{x}}_l}} \right)} \right|^2},\end{equation*}

where *l* is the index for voxels and ${N_{{\text{voxel}}}}$ is the number of voxels in the reference CBCT. On the other hand, the projection-domain loss function $L_{{\text{sim}}}^{{\text{prj}}}$ compared the DRRs with the corresponding cone-beam projections:

\begin{align*}L_{{\text{sim}}}^{{\text{prj}}} = \frac{1}{{{N_{{\text{batch}}}}\,{N_{{\text{pixel}}}}}}\mathop \sum \limits_{t \in batch} \mathop \sum \limits_{{N_{{\text{pixel}}}}} {\left| {\mathcal{P}\left[ {{\boldsymbol{I}}\left( {{\boldsymbol{x}},\,p} \right)} \right] - {\boldsymbol{p}}} \right|^2},\end{align*}

where ${N_{{\text{batch}}}}$ and ${N_{{\text{pixel}}}}$ respectively are the sample number in a batch and the pixel number of a projection, and $\mathcal{P}$ denotes the cone-beam projector that simulates DRRs from the dynamic CBCT ${\boldsymbol{I}}\left( {{\boldsymbol{x}},{\text{ }}p} \right)$. We set ${N_{{\text{batch}}}} = 32$ to balance the training speed and accuracy.

In addition to the similarity losses, a number of regularization losses were implemented to regularize the spatiotemporal reconstruction problem. The first regularization loss was the total variation (TV) loss on the spatial INR-reconstructed reference CBCT to suppress high-frequency image noise while preserving anatomy edges:

\begin{equation*}{L_{{\text{TV}}}} = \frac{1}{{{N_{{\text{voxel}}}}}}\mathop \sum \limits_l \left| {\nabla {{\boldsymbol{I}}_{{\text{ref}}}}\left( {{{\boldsymbol{x}}_l}} \right)} \right|,\end{equation*}

where $\nabla $ denotes the gradient operator. In addition, regularization losses were implemented to regularize the data-driven motion model. The second regularization loss promoted the ortho-normality of the MBCs:

\begin{equation*}{L_{MBC}} = \frac{1}{9}\mathop \sum \limits_{k = x,{\text{ }}y,{\text{ }}z} \sum\limits_{i = 1}^3 {\left( {{\left|{\left\| {{e_{i,k}}} \right\|}^2 { - 1} \right|^2} + \sum\limits_{j = i + 1}^3 {{{\left| {{e_{i,k}} \cdot {e_{j,k}}} \right|}^2}} } \right)} .\end{equation*}

The normalization constraint (i.e. the first term in the parenthesis in equation (6)) removes the ambiguity of the spatiotemporal decomposition equation (2) of the low-rank motion model, and the orthogonal constraint (i.e. the second term in the parenthesis in equation (6)) removes the ambiguity of the MBCs between different levels. The third regularization loss was the zero-mean score loss on the MBC scores ${{\boldsymbol{w}}_i}\left( p \right)$:

\begin{equation*}{L_{{\text{ZMS}}}} = \frac{1}{9}\mathop \sum \limits_{k = x,y,z} \mathop \sum \limits_{i = 1}^3 {\left| {\frac{1}{{{N_p}}}\mathop \sum \limits_p {w_{i,k}}\left( p \right)} \right|^2},\end{equation*}

where *N_p_* denotes the number of projections. The zero-mean score loss was enforced to remove the constant baseline of ${{\boldsymbol{w}}_i}\left( p \right)$.

Finally, a DVF self-consistency regularization loss was implemented. The self-consistency regularization enforces that the deformed reference CBCTs ${\boldsymbol{I}}\left( {{\boldsymbol{x}},{\text{ }}p} \right)$ followed by the inverse DVFs ${{\boldsymbol{d}}^{ - 1}}\left( {{\boldsymbol{x}},{\text{ }}p} \right)$ should return to the reference CBCT, which further conditions the ill-posed reconstruction problem:

\begin{equation*}{L_{{\text{SC}}}} = \frac{1}{{{N_{{\text{batch}}}}}}\mathop {\mathop \sum \nolimits }\limits_{t \in {\text{batch}}} \frac{1}{{{N_{{\text{voxel}}}}}}\mathop {\mathop \sum \nolimits }\limits_l {\left| {{\boldsymbol{I}}^0\left( {{{{\boldsymbol{x}}_l}},{\text{ }}p} \right) - {{\boldsymbol{I}}_{{\text{ref}}}}\left( {{{\boldsymbol{x}}_l}} \right)} \right|^2},\end{equation*}

where ${\boldsymbol{I}}^0\left( {\boldsymbol{x},{\text{ }}p} \right) = {\boldsymbol{I}}\left( {{\boldsymbol{x}} + {{\boldsymbol{d}}^{ - 1}}\left( {\boldsymbol{x},{\text{ }}p} \right),{\text{ }}p} \right)$. The inverse DVFs were calculated using the iterative algorithm (Chen *et al* 2008) with three iterations.

In summary, the first learning task of DREME is similar to PMF-STINR (Shao *et al* 2024), but with the temporal INR replaced by a CNN-based motion encoder. In addition, a zero-mean score loss on the motion coefficients and a DVF self-consistency regularization loss were added to further condition the learning. For more details on PMF-STINR, please refer to our previous publication (Shao *et al* 2024).

#### 2.1.4. Learning task 2: real-time motion estimation for real-time CBCT imaging

**Figure 4.**
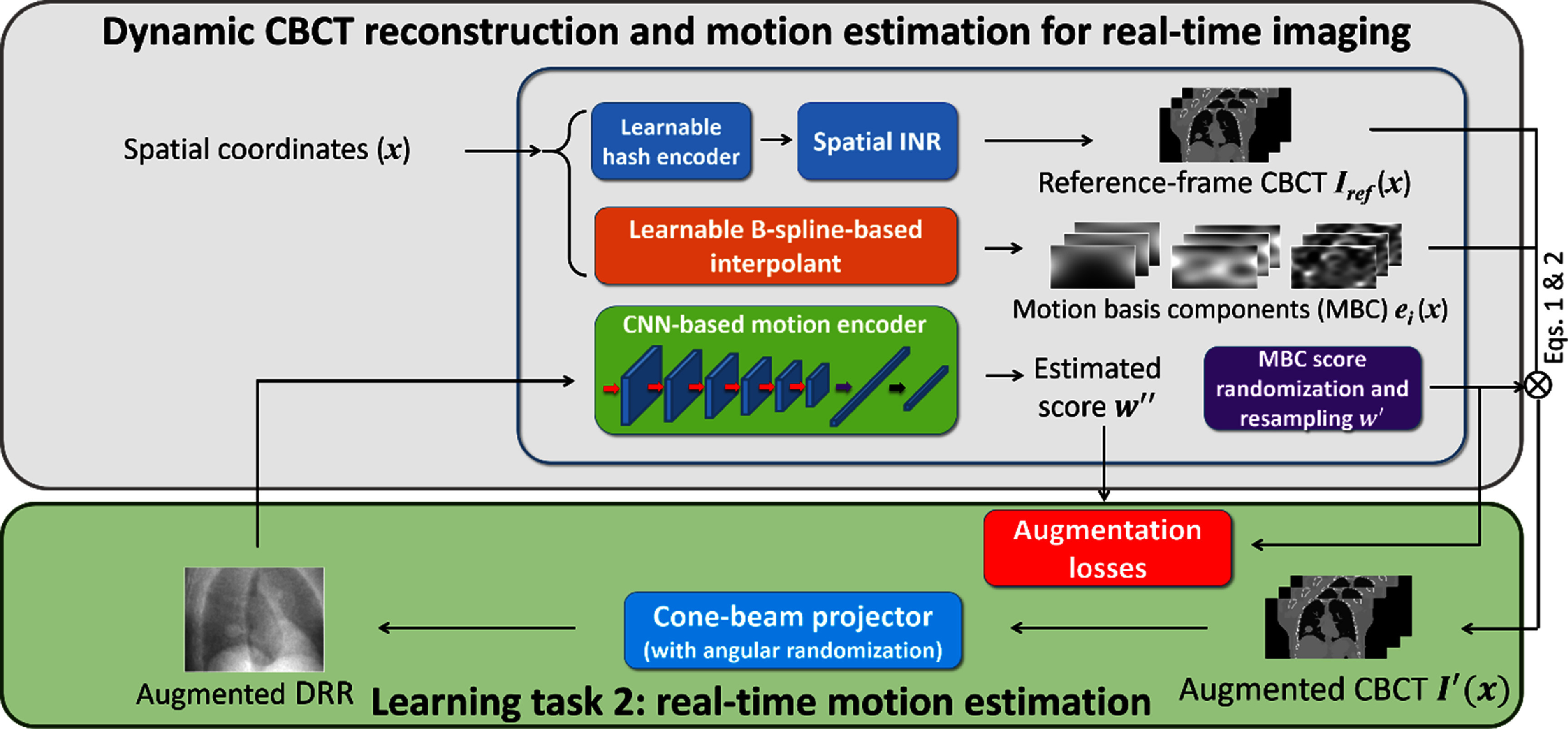
Learning task 2: real-time motion estimation. For the learning task of real-time imaging, deformable motion augmentation simulates random motion states by randomizing and resampling the MBC scores ${{\boldsymbol{w}}_i}\left( p \right)$ to enhance the model’s robustness to unseen motion states. In addition, projection angle augmentation is implemented to simulate DRRs of motion-augmented CBCTs at random projection angles, to promote angle-agnostic learning.

Although the CNN-based motion encoder is able to infer motion directly from cone-beam projections and can readily represent both pre-treatment dynamic motion and intra-treatment real-time motion, the motion encoder trained using only the pre-treatment CBCT projections ***p*** may simply learn to memorize the correspondence between the training projections and the corresponding scores as in the first learning task, and will not generalize to new, unseen motion observed in arbitrarily-angled intra-treatment projections. To train a motion- and angle-robust CNN, we need a second learning task to further enhance the CNN’s capability in real-time motion and CBCT estimation (figure 4). The second learning task further trains the CNN to estimate motion in real time from a single x-ray projection at an arbitrary projection angle, where the x-ray projection is augmented with motion different from that observed in the pre-treatment CBCT scan.

To simulate random deformable motion to further train the CNN, deformable augmentation was used to generate motion states unseen in the pre-treatment acquisition. The augmentation was implemented by randomly resampling the solved MBC scores ${w_{i,k}}\left( p \right)$ from the dynamic CBCT reconstruction for each projection, using the following equation:

\begin{equation*}w_{i,k}^{\prime}\left( p \right) = {r_1}\left( p \right) \times {r_2}\left( {i,k} \right) \times {w_{i,k}}\left( p \right),\end{equation*}

where ${r_1} \in \left[ {0.6,{\text{ }}2.0} \right]$ and ${r_2} \in \left[ {0.8,{\text{ }}1.2} \right]$ are uniformly distributed random variables. Two random variables were used to instill independent randomness between different levels *i* and Cartesian directions *k* (through the level/direction-specific factors ${r_2}\left( {i,k} \right)$), while still maintaining a certain degree of motion correlation among the MBC scores of each instance (through the same overall scaling factor ${r_1}$ (*p*) for each projection). Based on the augmented MBC scores, the DVFs are randomized (equation (2)), resulting in various deformation-augmented CBCTs (equation (1)). From the deformation-augmented CBCTs, we generated on-the-fly DRRs using a cone-beam projector during the training, and input them into the motion encoder CNN to infer the MBC scores $w_{{i},{k}}^{^{\prime}\;\; ^{\prime}}\left( p \right)$. The augmentation loss was then defined as

\begin{equation*}{L_{{\text{Aug}}}} = \frac{1}{{9{N_{{\text{batch}}}}}}\mathop \sum \limits_{t \in {\text{batch}}} \mathop \sum \limits_{k = x,y,z} \mathop \sum \limits_{i = 1}^3 {\left| {w_{{i},{k}}^{^{\prime}\;\; ^{\prime}}\left( p \right) - w_{i,k}^{\prime}\left( p \right)} \right|^2}.\end{equation*}

During the network training, we avoided using intermediate motion models still in training for deformation augmentation to prevent instabilities (table 1). Instead, we only re-generated DRRs at random angles (angle augmentation only) from the solved dynamic CBCTs, and fed them into the CNN motion encoder to infer the MBC scores to compute the loss in equation (10). The deformation augmentation was added in stage II-d, after the motion model was trained and frozen (table 1).

**Table 1.** The progressive multi-resolution training strategy with different loss functions. The symbol ✓ indicates that the corresponding loss function was used in the specific training stage.

Training stage		I-a	I-b	I-c	II-a	II-b	II-c	II-d
Number of epochs		400	700	1700	1000	1000	1000	1000

Spatial resolution (mm^3^)		4 × 4 × 4			2 × 2 × 2			

Spatial INR learning rate		4 × 10^−4^	4 × 10^−5^	1 × 10^−5^	1 × 10^−3^	4 × 10^−4^	1 × 10^−4^	0.
Motion encoder learningrate		0.	0.	1 × 10^−3^	0.	0.	1 × 10^−4^	1 × 10^−4^
B-spline interpolant learning rate		0.	0.	1 × 10^−3^	0.	0.	1 × 10^−4^	0.

Loss function	Weighting factor *λ*							
Image-domain similarity loss equation (3)	1.	✓			✓			

Projection-domain similarity loss equation (4)	1.		✓	✓		✓	✓	

TV regularization equation (5)	2 × 10^−4^		✓	✓		✓	✓	

MBC regularization equation (6)	1.			✓			✓	

Zero-mean score regularization equation (7)	1 × 10^−3^			✓			✓	

DVF self-consistency regularization equation (8)	1 × 10^3^			✓			✓	

Motion/angle augmentation loss equation (10)	1 × 10^−4^					✓ (angle augmentation only)	✓ (angle augmentation only)	✓ (motion + angle augmentation)

### 2.2. Onboard real-time CBCT imaging and target localization

**Figure 5.**
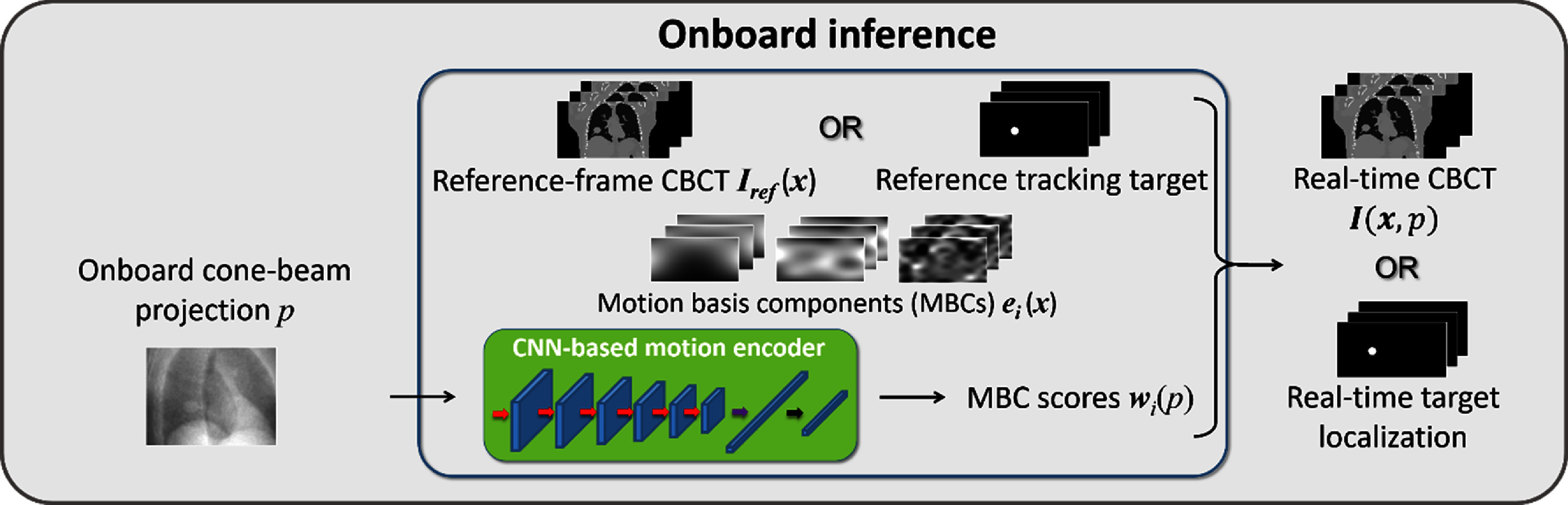
Onboard inference stage for real-time CBCT imaging and target localization. During the onboard inference stage, the CNN-based motion encoder takes an onboard projection p at an arbitrary angle as input, and estimates the MBC scores ${{\boldsymbol{w}}_i}\left( p \right)$ to derive real-time CBCT ${\boldsymbol{I}}\left( {{\boldsymbol{x}},{\text{ }}p} \right)$ or 3D target.

Through dual-task learning, DREME reconstructs the latest patient 3D anatomy and patient-specific motion model while simultaneously using the CNN motion encoder to learn the correlation between the MBC scores ${{\boldsymbol{w}}_i}\left( p \right)$ and the motion-related image features embedded in an arbitrarily-angled cone-beam projection *p*, making it readily deployable for online real-time CBCT imaging and target localization (figure 5). For real-time CBCT imaging, the real-time CBCT volume ${\boldsymbol{I}}\left( {{\boldsymbol{x}},{\text{ }}p} \right)$ is generated by deforming the reference CBCT ${{\boldsymbol{I}}_{{\text{ref}}}}\left( {\boldsymbol{x}} \right)$ using the motion field ${\boldsymbol{d}}\left( {{\boldsymbol{x}},{\text{ }}p} \right)$ estimated from the projection *p* via the CNN-based motion encoder. For real-time target localization, the reference CBCT can be replaced by the contour of the tracking target (represented by a binary mask) delineated from the reference CBCT to achieve real-time target localization. We note that the reference CBCT and motion model are specific to the current treatment session and may not directly apply to new treatment sessions or new patients. However, for the same patient, the motion/anatomy prior learned from a treatment session can be used to initialize the learning in subsequent sessions to accelerate the training.

#### 2.3. A progressive training strategy

For DREME training, we followed our previous works (Zhang *et al* 2023, Shao *et al* 2024) by designing a progressive multi-resolution training strategy to improve learning efficiency and avoid overfitting. The strategy progressively increases the learning complexity under two spatial resolutions (table 1). The total loss function ${L_{{\text{tot}}}}$ was a weighted sum of the aforementioned loss functions, with corresponding weighting factors specified in table 1. Examples of the evolution of the loss functions and intermediate network outputs during the progressive training scheme were provided in section I of supplementary materials to illustrate the training dynamics of DREME.

Remarks on the training strategy are in order: **(a)** The low-resolution reconstruction contained the training stage I with an isotropic 4 × 4 × 4 mm^3^ resolution (for intermediate CBCT inference from the spatial INR), and the resolution was doubled at the stage II (2 × 2 × 2 mm^3^). Due to the GPU memory limits, we were unable to proceed to a higher resolution, though this constraint is not a fundamental limitation of the proposed framework. **(b)** Both low- and high-resolution stages followed a parallel training structure that each started from the image-domain similarity loss (equation (3)) and then switched to projection-domain similarity loss (equation (4)). The motion encoder and the B-spline interpolant entered the training at later sub-stages (I-c and II-c). **(c)** The approximate CBCT ${{\boldsymbol{I}}_{{\text{app}}}}\left( x \right)$ at low-resolution training was obtained from the Feldkamp–Davis–Kress reconstruction (Feldkamp *et al* 1984) of all the x-ray projections within $\boldsymbol{p}$. At the stage of the high-resolution reconstruction, it was obtained by the tri-linear up-sampling from the solved ${{\boldsymbol{I}}_{{\text{ref}}}}\left( {\boldsymbol{x}} \right)$ by the low-resolution training stage. **(d)** Zero learning rate meant the learnable parameters in the corresponding network component were frozen. **(e)** The second learning task (figure 4) was introduced at stage II (from II-b). When the spatial INR and the motion model were still in training (non-zero learning rates), we did not perform deformation augmentation, and only re-generated DRRs from the pre-treatment x-ray projections with new, random angles (angle augmentation only) to train the CNN motion encoder. The last stage (stage II-d) was dedicated to the second learning task with both motion and angle augmentation enabled, and the spatial INR and the B-spline motion model frozen (learning rates set to 0). **(f)** The values of the weighting factors were determined by empirical searching.

### 2.4. Evaluation datasets and schemes

We evaluated DREME using the extended cardiac torso (XCAT) digital phantom (Segars *et al* 2010) and a multi-institutional lung patient dataset. The XCAT simulation study provided ‘ground truth’ for quantitative evaluation, thus allowing us to assess the model and training strategy designs and optimize network hyper-parameters. The patient study allowed us to evaluate the clinical application potential of DREME. Given the different natures of the two datasets, we discuss them separately in the following subsections.

#### 2.4.1. XCAT simulation study

**Figure 6.**
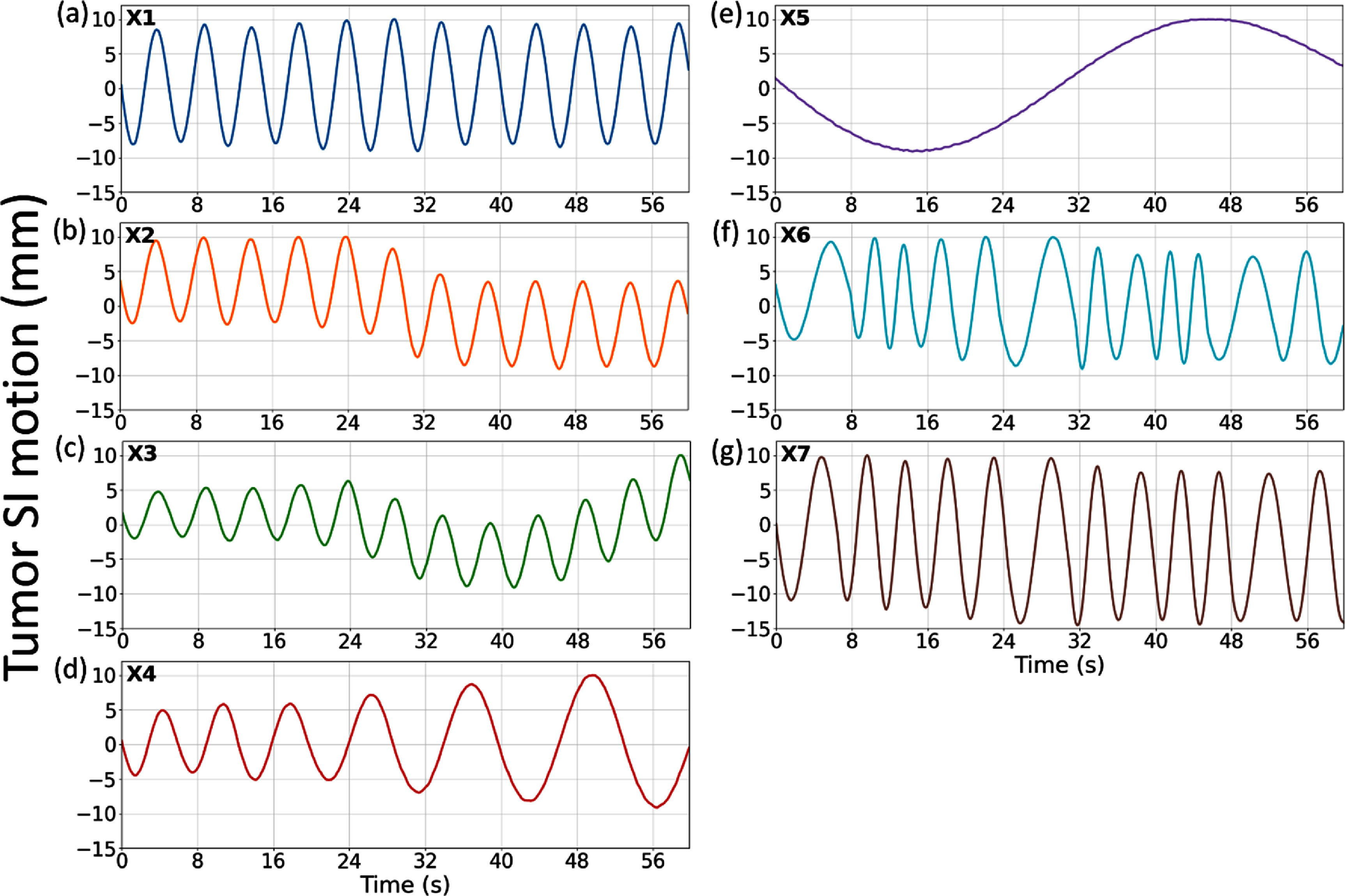
Lung tumor motion trajectories along the superior-inferior (SI) direction in the XCAT simulation study. Trajectories X1–X6 shared the same maximum motion ranges in the SI direction, whereas the SI range of X7 was extended to evaluate the robustness of DREME models trained using the other scenarios (X1–X6).

We simulated an XCAT phantom to cover the thorax and upper abdomen region, with a volume of 200 × 200 × 100 voxels and a 2 × 2 × 2 mm^3^ voxel size. A spherical lung tumor with a 30 mm diameter was inserted in the lower lobe of the right lung as a motion tracking target. Seven respiratory motion trajectories (X1–X7) were simulated (figure 6), featuring motion scenarios combining variations in breathing amplitudes, frequencies, and/or patterns. Different motion trajectories were used to evaluate DREME’s generalizability to different motion scenarios, especially when there is a discrepancy between the training and testing scenarios. After generating XCAT volumes for these scenarios, we simulated x-ray projections ${\boldsymbol{p}}$ from them as pre-treatment cone-beam scans. The projections were generated in full-fan mode, including 660 projections covering a 360° gantry angle. The scan time was 60 s (with 11 frames per second). Each projection spanned 256 × 192 pixels with a 1.55 × 1.55 mm^2^ pixel resolution. In addition to the pre-treatment scans, we also simulated projections on each motion scenario to serve as intra-treatment scans to test the real-time imaging capability of DREME. To test the angle-agnostic capability of the trained CNN motion encoder, the second sets of cone-beam projections were simulated with the gantry angle rotated by 90.27° with respect to the pre-treatment sets, to ensure that no two x-ray projections in the training and testing sets share the same motion state and the projection angle. We used the tomographic package ASTRA toolbox to simulate the cone-beam projections (van Aarle *et al* 2016). The same toolbox was also used in the DREME network as the cone-beam projector.

As a ‘one-shot’ learning technique, DREME was separately trained on each of the first six motion scenarios (X1–X6) and tested on all scenarios for real-time motion estimation (X1–X7), yielding six groups of results. This cross-scenario testing evaluates the robustness of DREME to testing scenarios very different from the training (figure 6). The performance of DREME was evaluated based on the image quality of the solved real-time CBCTs ${\boldsymbol{I}}\left( {{\boldsymbol{x}},{\text{ }}p} \right)$ and the accuracy of lung tumor localization. The image quality was evaluated via the mean relative error metric (RE):

\begin{align*}{\text{RE}} = \frac{1}{{{N_p}}}\mathop {\mathop \sum \nolimits }\limits_p \sqrt {\frac{{\mathop \sum \nolimits _{l = 1}^{{N_{{\text{voxel}}}}}||I\left( {{\boldsymbol{x}_l},{\text{ }}p} \right) - {I^{gt}}{{\left( {{\boldsymbol{x}_l},{\text{ }}p} \right)}||^2}}}{{\mathop \sum \nolimits _{l = 1}^{{N_{{\text{voxel}}}}}||{I^{gt}}{{\left( {{\boldsymbol{x}_l},{\text{ }}p} \right)}||^2}}}} ,\end{align*}

where ${{\boldsymbol{I}}^{gt}}\left( {{\boldsymbol{x}},{\text{ }}p} \right)$ is the ‘ground-truth’ CBCT. The structural similarity index measure (SSIM) was also evaluated between the reconstructed and ‘ground-truth’ real-time CBCTs. The tumor localization accuracy was evaluated by contour-based metrics, including the tumor center-of-mass error (COME) and Dice similarity coefficient (DSC). We contoured the lung tumor from the reconstructed reference CBCTs, and then propagated the reference tumor contours to other instances using solved real-time DVFs and compared with the ‘ground-truth’ contours.

##### 2.4.2. Patient study

The patient dataset included 16 patients from three institutes. Three patients (P1–P3) were from the MD Anderson Cancer Center (Lu *et al* 2007), six patients (P9 and P12–P16) from the UT Southwestern Medical Center (Shao *et al* 2024), and seven patients (P4–P8, P10–P11) from the SPARE challenge dataset (Shieh *et al* 2019). All scans covered the thoracic region, except for P8, whose scan covered the abdominal region. These patients were selected with observed respiration-induced motion in the thoracic-abdominal region. The scans were under various acquisition protocols including full-/half-fan scans, different kVp/mA/ms, and varying cone-beam geometries (i.e. source-to-axis distance/source-to-detector distance) etc. The acquisition protocols, sizes of the CBCT volumes, and other details of the dataset are summarized in table 2. As all cases had only a single pre-treatment scan, the cone-beam projections were divided into a training set and a testing set. For P1–P3 and P12–P16, every third projection from their pre-treatment scan was selected for model training, and the remaining two-thirds of the projections were used for model testing. For P9, due to limited projection numbers, every second projection was selected for training, and the remaining half of the projection set was used for testing. For the SPARE challenge patients (P4–P8, P10–P11), the training dataset already included down-sampled scans for the spare-view reconstruction challenge (Shieh *et al* 2019). Thus, we trained DREME on the down-sampled scans and tested it on the fully-sampled sets, excluding the projections already in the training sets.

**Table 2.** CBCT imaging and reconstruction parameters for the multi-institutional patient dataset.

Patient ID	Source^a^	Vender	Scan mode	Projection size^b^	Pixel size (mm^2^)	kVp/mA/ms	SAD^c^ (mm)/SDD (mm)	Reconstructed CBCT voxels	Voxel size (mm^3^)
P1	MDACC	Varian	Full fan	512 × 384 × 1983	0.776 × 0.776	120/80/25	1000/1500	200 × 200 × 100	2 × 2 × 2
P2	MDACC	Varian	Full fan	512 × 384 × 2729	0.776 × 0.776	120/80/25	1000/1500	200 × 200 × 100	2 × 2 × 2
P3	MDACC	Varian	Full fan	512 × 384 × 1653	0.776 × 0.776	120/80/25	1000/1500	200 × 200 × 100	2 × 2 × 2
P4	SPARE	Elekta	Full fan	512 × 512 × 1015	0.8 × 0.8	125/20/20	1000/1536	200 × 200 × 100	2 × 2 × 2
P5	SPARE	Elekta	Full fan	512 × 512 × 1005	0.8 × 0.8	125/20/20	1000/1536	200 × 200 × 100	2 × 2 × 2
P6	SPARE	Elekta	Full fan	512 × 512 × 1012	0.8 × 0.8	125/20/20	1000/1536	200 × 200 × 100	2 × 2 × 2
P7	SPARE	Elekta	Full fan	512 × 512 × 1016	0.8 × 0.8	125/20/20	1000/1536	200 × 200 × 100	2 × 2 × 2
P8	SPARE	Elekta	Full fan	512 × 512 × 1007	0.8 × 0.8	125/20/20	1000/1536	200 × 200 × 100	2 × 2 × 2
P9	UTSW	Varian	Full fan	1024 × 768 × 500	0.388 × 0.388	80/20/10	1000/1500	200 × 200 × 100	2 × 2 × 2
P10	SPARE	Varian	Half fan	1006 × 750 × 2416	0.388 × 0.388	120/20/20	1000/1500	300 × 300 × 102	2 × 2 × 2
P11	SPARE	Varian	Half fan	1006 × 750 × 2918	0.388 × 0.388	120/20/20	1000/1500	300 × 300 × 102	2 × 2 × 2
P12	UTSW	Varian	Half fan	1024 × 768 × 895	0.388 × 0.388	125/15/20	1000/1500	310 × 310 × 102	2 × 2 × 2
P13	UTSW	Varian	Half fan	1024 × 768 × 895	0.388 × 0.388	125/15/20	1000/1500	300 × 300 × 102	2 × 2 × 2
P14	UTSW	Varian	Half fan	1024 × 768 × 894	0.388 × 0.388	125/60/20	1000/1500	300 × 300 × 102	2 × 2 × 2
P15	UTSW	Varian	Half fan	1024 × 768 × 894	0.388 × 0.388	125/15/20	1000/1500	300 × 300 × 102	2 × 2 × 2
P16	UTSW	Varian	Half fan	1024 × 768 × 894	0.388 × 0.388	125/15/20	1000/1500	300 × 300 × 102	2 × 2 × 2

^a^ MDACC: MD Anderson Cancer Center (Lu *et al* 2007). SPARE: SPARE Challenge (Shieh *et al* 2019). UTSW: University of Texas Southwestern Medical Center.

^b^ Width (in pixel number) × height (in pixel number) × *N*_p_ (number of projections).

^c^ SAD: source-to-axis distance. SDD: source-to-detector distance.

As ‘ground-truth’ data were unavailable for the patient study, to evaluate the accuracy of motion estimation, we re-projected the reconstructed CBCT volumes into 2D DRRs and compared them with the acquired projections in the testing set, via motion features tracked by the Amsterdam Shroud (AS) method (Zijp *et al* 2004). Details of the AS method were provided in our previous work (Shao *et al* 2024). In brief, the intensity gradients along the vertical axis (i.e. the superior-inferior (SI) direction) were calculated for both cone-beam projections and DRRs to highlight anatomical landmarks with high contrast edges (e.g. diaphragm). These intensity gradients were integrated along the horizontal axis over a region of interest that exhibited clear motion-induced intensity variations to form an AS image. Finally, the AS images were post-processed to enhance their contrast for motion trace extraction, and the localization accuracy was calculated by the difference between the extracted traces from the cone-beam projections and from the DRRs. In addition, Pearson correlation coefficients between the extracted traces were calculated.

### 2.5. Comparison and ablative studies

We compared DREME with a principal component analysis (PCA)-based 2D3D deformable registration technique (Li *et al* 2011, Shao *et al* 2022) on the XCAT dataset. The 2D3D registration solves a 3D DVF by matching DRRs of the deformed/registered anatomy with acquired x-ray projections. This technique incorporates patient-specific prior knowledge to alleviate the instability of the inverse problem. We used PCA-based motion models for the 2D3D registration. Two variants of the motion models were developed, depending on the sources from which the motion models were derived. The PCA motion model can be derived from a 4D-CT scan during treatment planning, which can potentially introduce biases (section 1). Alternatively, the motion model can be directly derived from the pre-treatment cone-beam scan (4D-CBCT). However, 4D reconstruction requires motion sorting/binning of the projections according to the respiratory phases, typically resulting in an under-sampled reconstruction. We sorted the pre-treatment scan projections into 10 respiratory phases and obtained inter-phase DVFs by registering the 4D-CBCT to the end-of-exhale phase CBCT, and then performed PCA on the inter-phase DVFs to derive principal motion components for the 2D3D deformable registration. DREME was compared with both variants, which were called 2D3D_PCA-4DCT_ and 2D3D_PCA-4DCBCT_, respectively, according to the source of the PCA motion model. Wilcoxon signed-rank tests were used to evaluate the significance level of observed differences.

As discussed in section 2.1.3 and shown in table 1, a number of loss functions were used to regularize the inverse problem and achieve a stable motion model. To investigate the benefits and roles of these regularization terms, an ablation study was conducted on the XCAT dataset by removing different loss functions in the training process. We removed the zero-mean score loss ${L_{{\text{ZMS}}}}$, DVF self-consistency loss ${L_{{\text{SC}}}}$, motion/angle augmentation loss (${L_{{\text{Aug}}}}$), and evaluated the impacts on the image quality and motion tracking accuracy. These variants of the DREME framework in the ablation study are called DREME_WO-ZMS_, DREME_WO-SC_, and DREME_WO-Aug_, where the subscript indicates which loss functions were removed. Wilcoxon signed-rank tests were used to evaluate the significance level of observed differences.

## 3. Results

### 3.1. The XCAT study results

**Figure 7.**
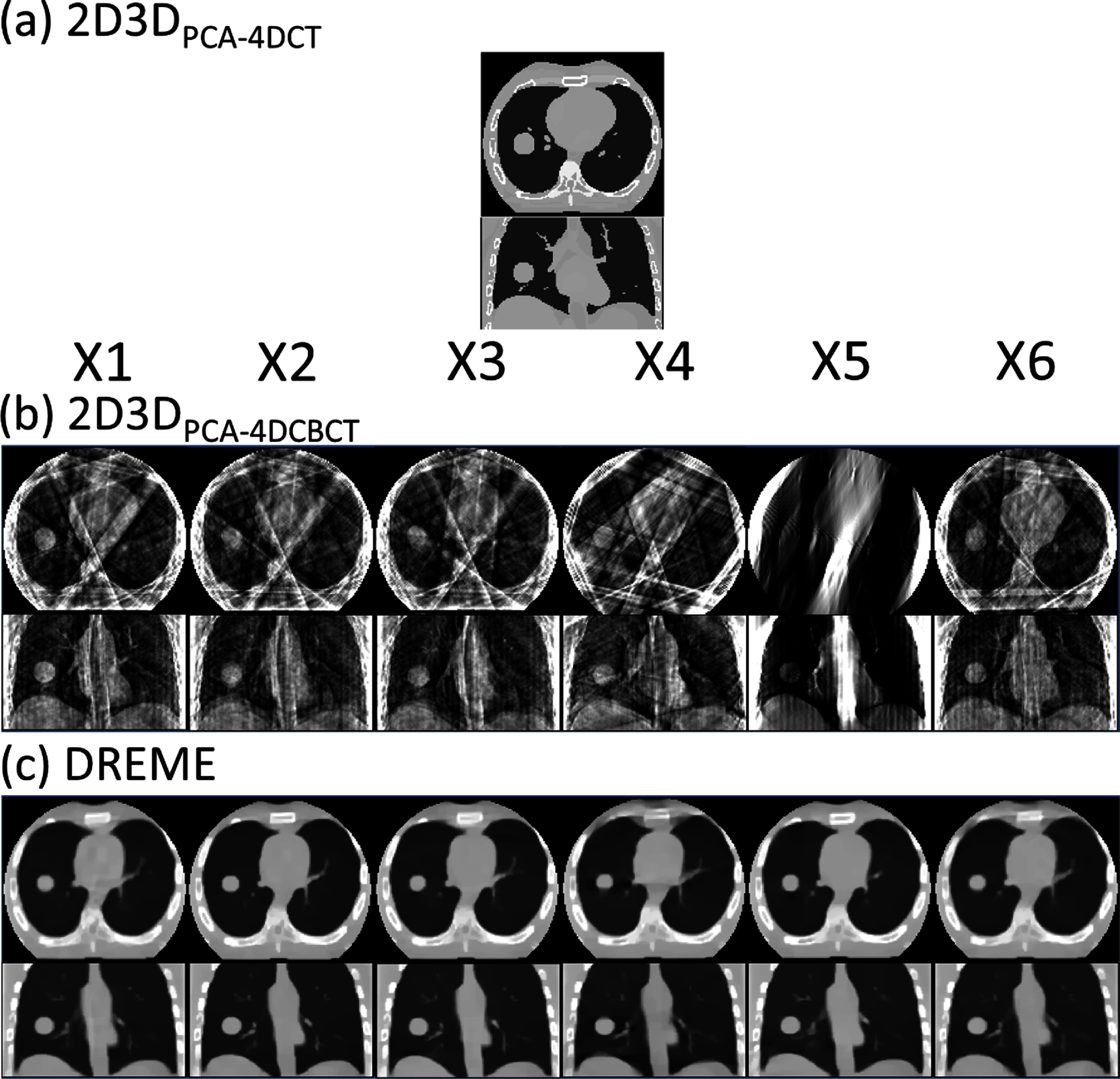
Comparison of reconstructed reference CBCTs in the comparison study. 2D3D_PCA-4DCT_ assumed the existence of an artifact-free pre-treatment 4D-CT scan and used the end-of-exhale phase as the reference anatomy. 2D3D_PCA-4DCBCT_ and DREME reconstructed the reference CBCTs using the pre-treatment scans, thus the anatomy is up-to-date and specific to daily motion scenarios. The reference CBCTs of 2D3D_PCA-4DCT_ were reconstructed from the end-of-exhale phase after projection phase sorting, suffering from significant under-sampling and motion-related artifacts. DREME reconstructed the reference CBCTs with simultaneous motion estimation/compensation, resulting in minimal motion-related artifacts.

Figure 7 compares the reference CBCTs used in the 2D3D-based methods and those reconstructed by DREME for the six motion scenarios (X1–X6) in the axial and coronal views. The reference CBCTs of 2D3D_PCA-4DCT_ and 2D3D_PCA-4DCBCT_ correspond to the end-of-exhale phase. 2D3D_PCA-4DCT_ used the end-of-exhale phase image from a prior 4D-CT scan, and the 4D-CT-derived PCA motion model was used in 2D3D deformable registrations for all motion scenarios (X1–X6). For 2D3D_PCA-4DCBCT_, the reference CBCTs were reconstructed from the phase-sorted pre-treatment scans, which were severely affected by under-sampling artifacts (∼66 projections for the end-of-exhale phase). The X5 scenario of slow breathing (or, equivalently, fast gantry rotation) also suffered from the issue of limited-angle reconstruction, as the end-of-exhale projections were confined to a narrow range of projection angles. These artifacts posed substantial challenges for the 2D3D-based registration technique.

Tables 3 and 4 respectively summarize the mean RE and mean SSIM from the comparison and ablation studies. All Wilcoxon signed-rank tests between DREME and other methods yielded p values < 10^−3^. DREME outperformed the 2D3D-based techniques in terms of both RE and SSIM. On the other hand, for the ablation study, DREME offered the best results among all, demonstrating the benefits of including various loss functions to condition the real-time imaging problem, especially the motion/angle augmentation losses used in the learning task 2.

**Table 3.** Mean relative error of the XCAT simulation study. The results for each training scenario (X1–X6) are presented as the mean and standard deviation (Mean ± SD), averaged over all testing scenarios (X1–X7).

	Comparison study		Ablation study			
Training scenario	2D3D_PCA-4DCT_	2D3D_PCA-4DCBCT_	DREME_WO-ZMS_	DREME_WO-SC_	DREME_WO-Aug_	DREME
X1	0.192 ± 0.050	0.575 ± 0.053	0.164 ± 0.014	**0.162 ± 0.016**	0.198 ± 0.047	**0.162 ± 0.015**
X2		0.591 ± 0.066	0.166 ± 0.016	0.164 ± 0.016	0.214 ± 0.056	**0.158 ± 0.018**
X3		0.617 ± 0.067	0.169 ± 0.022	0.168 ± 0.020	0.223 ± 0.060	**0.165 ± 0.022**
X4		0.732 ± 0.074	0.191 ± 0.015	0.193 ± 0.017	0.243 ± 0.052	**0.185 ± 0.015**
X5		1.328 ± 0.379	0.173 ± 0.021	0.177 ± 0.021	0.261 ± 0.049	**0.172 ± 0.019**
X6		0.562 ± 0.070	**0.169 ± 0.016**	0.170 ± 0.014	0.215 ± 0.053	0.170 ± 0.015

**Table 4.** Mean SSIM of the XCAT simulation study. The results for each training scenario (X1–X6) are presented as the mean and standard deviation (Mean ± SD), averaged over all testing scenarios (X1–X7).

	Comparison study		Ablation study			
Training scenario	2D3D_PCA-4DCT_	2D3D_PCA-4DCBCT_	DREME_WO-ZMS_	DREME_WO-SC_	DREME_WO-Aug_	DREME
X1	0.9777 ± 0.0109	0.8500 ± 0.0130	0.9810 ± 0.0031	0.9813 ± 0.0035	0.9728 ± 0.0122	**0.9815 ± 0.0033**
X2		0.8477 ± 0.0161	0.9804 ± 0.0035	0.9808 ± 0.0035	0.9684 ± 0.0145	**0.9819 ± 0.0039**
X3		0.8433 ± 0.0131	0.9795 ± 0.0051	0.9795 ± 0.0048	0.9663 ± 0.0161	**0.9803 ± 0.0053**
X4		0.8029 ± 0.0127	0.9743 ± 0.0039	0.9737 ± 0.0047	0.9596 ± 0.0154	**0.9759 ± 0.0038**
X5		0.6027 ± 0.0165	**0.9784 ± 0.0046**	0.9770 ± 0.0046	0.9556 ± 0.0138	**0.9784 ± 0.0042**
X6		0.8549 ± 0.0164	**0.9794 ± 0.0036**	0.9793 ± 0.0032	0.9678 ± 0.0142	0.9791 ± 0.0033

Tables 5 and 6 respectively summarize the mean tumor COME and DSC results. All Wilcoxon signed-rank tests of COME between DREME and the other methods yielded *p* values < 10^−3^, except for DREME_WO-ZMS_ (*p* = 0.007), and all Wilcoxon signed-rank tests of DSC between DREME and the other methods yielded *p* values < 10^−3^. Even with a high-quality reference CBCT, it remained challenging for 2D3D_PCA-4DCT_ to locate the tumors accurately. We found the error generally increased when the respiratory phase of real-time CBCTs moved away from the PCA reference CBCTs (i.e. end-of-exhale phase), which indicates that the 2D3D-based registration algorithm may have been trapped at local minima when estimating motion with large amplitudes. 2D3D_PCA-4DCBCT_ had very poor localization performance, as the reconstruction artifacts in the reference CBCTs propagated to the tumor localization errors. Overall, DREME achieved sub-voxel COME.

**Table 5.** Lung tumor center-of-mass error (COME, in mm) of the XCAT simulation study. The results of each training scenario show the mean and standard deviation (Mean ± SD) averaged over all testing scenarios (X1–X7).

	Comparison study		Ablation study			
Training scenario	2D3D_PCA-4DCT_	2D3D_PCA-4DCBCT_	DREME_WO-ZMS_	DREME_WO-SC_	DREME_WO-Aug_	DREME
X1	3.1 ± 3.5	7.0 ± 4.4	1.0 ± 0.7	1.0 ± 0.9	3.6 ± 3.4	**0.9 ± 0.8**
X2		7.6 ± 4.6	1.1 ± 0.8	1.6 ± 0.8	5.0 ± 3.8	**1.0 ± 0.7**
X3		8.4 ± 4.7	**1.2 ± 0.9**	1.3 ± 1.0	5.5 ± 4.2	**1.2 ± 1.0**
X4		10.0 ± 5.0	**1.6 ± 1.1**	1.9 ± 0.9	6.3 ± 4.1	1.7 ± 0.9
X5		15.4 ± 6.7	**1.3 ± 1.1**	1.5 ± 1.2	7.5 ± 4.0	**1.3 ± 1.0**
X6		9.6 ± 4.7	**1.2 ± 0.9**	1.2 ± 0.9	4.9 ± 3.9	1.3 ± 0.8

**Table 6.** Lung tumor Dice similarity coefficient (DSC) of the XCAT simulation study. The results of each training scenario show the mean and standard deviation (Mean ± SD) averaged over all testing scenarios (X1–X7).

	Comparison study		Ablation study			
Training scenario	2D3D_PCA-4DCT_	2D3D_PCA-4DCBCT_	DREME_WO-ZMS_	DREME_WO-SC_	DREME_WO-Aug_	DREME
X1	0.850 ± 0.135	0.640 ± 0.144	0.918 ± 0.028	**0.922 ± 0.034**	0.812 ± 0.147	0.919 ± 0.029
X2		0.590 ± 0.124	0.902 ± 0.029	0.920 ± 0.029	0.757 ± 0.166	**0.922 ± 0.028**
X3		0.596 ± 0.159	0.900 ± 0.032	**0.913 ± 0.037**	0.731 ± 0.181	0.910 ± 0.035
X4		0.570 ± 0.133	0.861 ± 0.035	0.881 ± 0.033	0.692 ± 0.178	**0.884 ± 0.032**
X5		0.334 ± 0.184	0.895 ± 0.042	0.885 ± 0.045	0.641 ± 0.175	**0.888 ± 0.037**
X6		0.478 ± 0.178	0.892 ± 0.029	**0.910 ± 0.032**	0.754 ± 0.169	0.902 ± 0.031

**Figure 8.**
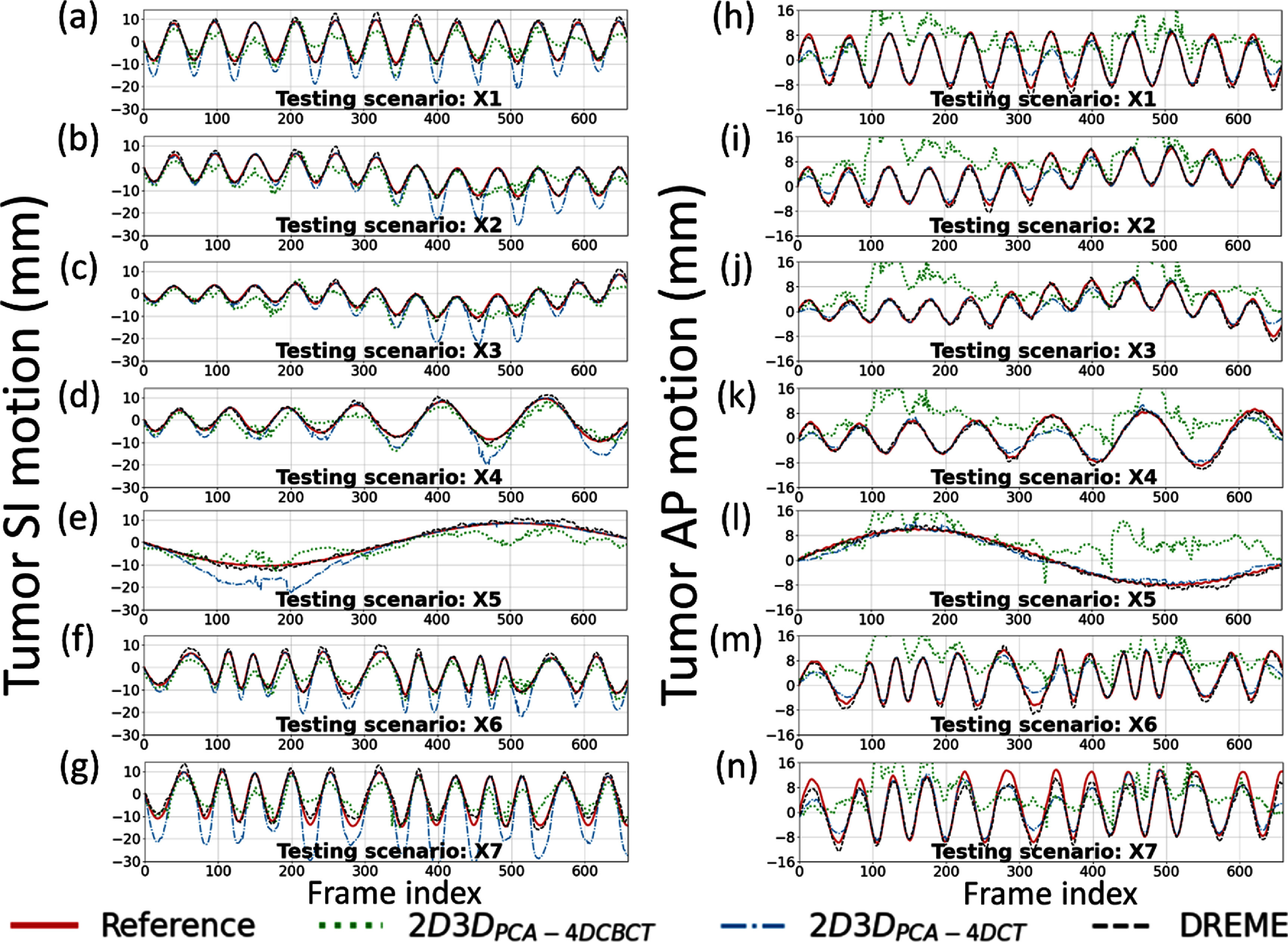
Tumor motion trajectories of the comparison study. The first and second columns respectively presents the comparison of the solved tumor motion for the X1–X7 scenarios in the superior-inferior (SI) and anterior-posterior (AP) directions between 2D3D_PCA-4DCBCT_, 2D3D_PCA-4DCT_, DREME, and the ‘ground-truth’ reference. DREME was trained on the X3 scenario.

**Figure 9.**
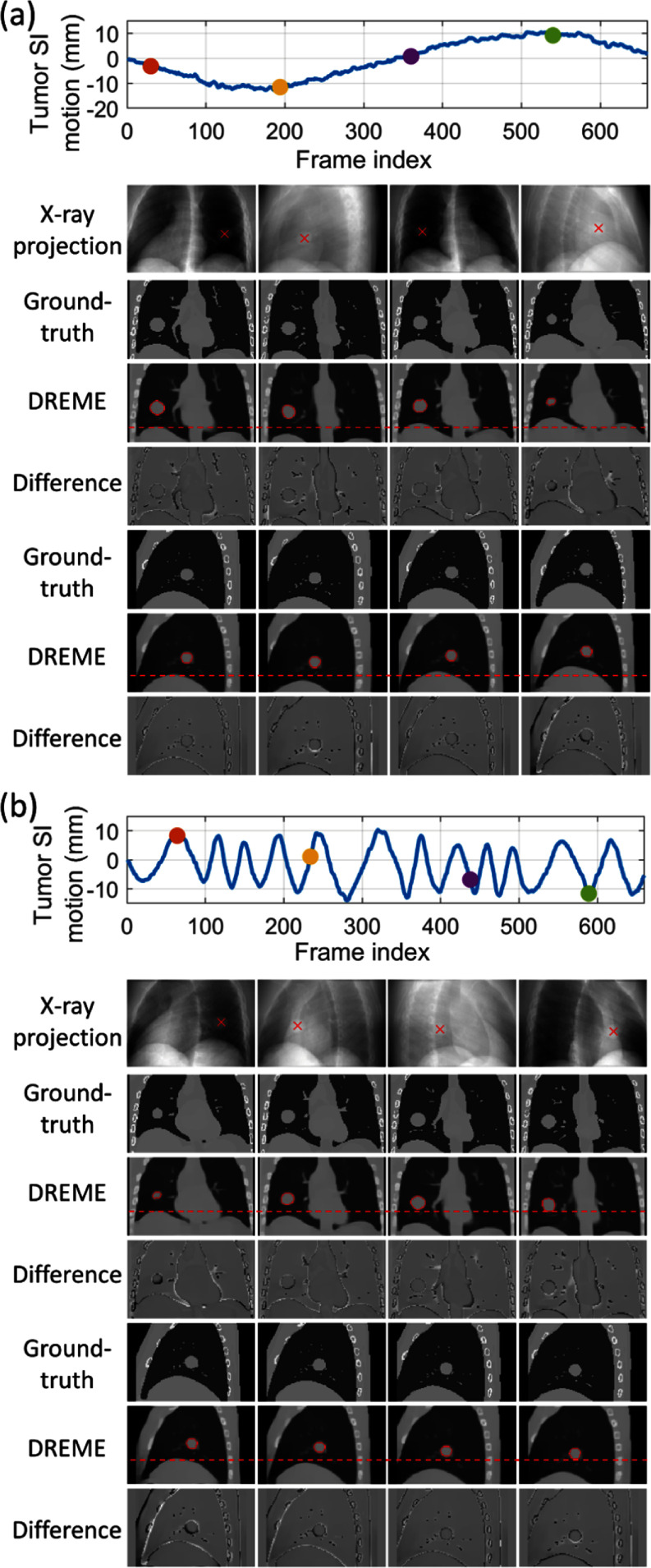
Examples of real-time CBCTs and lung tumor motion solved by DREME for the XCAT study: (a) X5 scenario and (b) X6 scenario. The training scenario was X3. The first row shows the tumor motion curves along the SI direction, with the dots indicating the time points selected for plotting. The second row presents the onboard x-ray projections at the selected time points, and the ‘×’ symbols indicate the solved tumor center-of-mass positions projected onto the projections. In the following rows, real-time CBCTs of the selected time points are compared against the ‘ground-truth’ CBCTs, with the difference images calculated. The estimated tumor contours (red) are also presented for each selected time points.

Figure 8 presents the solved tumor motion trajectories in the comparison study. In this figure, DREME was trained on the X3 scenario and tested across all scenarios (X1–X7). Overall, DREME showed more accurate and stable performance. In contrast, 2D3D_PCA-4DCT_ exhibited significant deviations in the SI direction, particularly near the end-of-inhale phase. Additionally, 2D3D_PCA-4DCBCT_ showed the most unstable tracking accuracy, due to its low-quality motion models. Figure 9 presents two scenarios (X5 and X6) of solved real-time CBCTs with tumor contours in the coronal and sagittal views and compares them with the corresponding ‘ground-truth’ CBCTs. The training scenario was X3. The real-time CBCTs solved by DREME matched well with the ‘ground truth’.

### 3.2. The patient study results

**Figure 10.**
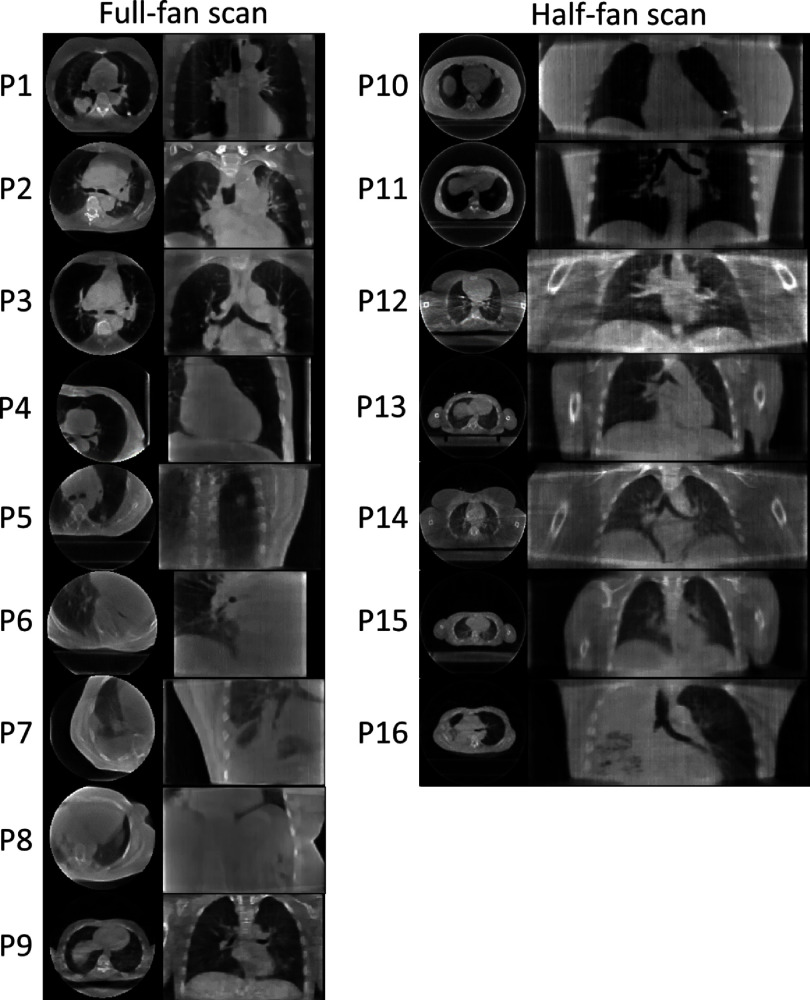
Reference CBCTs reconstructed by DREME for the patient study. Left and right columns present the axial and coronal views of the reference CBCTs for the full-fan and half-fan scans, respectively. Except for P8, whose CBCT scan covered the abdominal region, the other CBCT scans covered the thoracic region.

**Figure 11.**
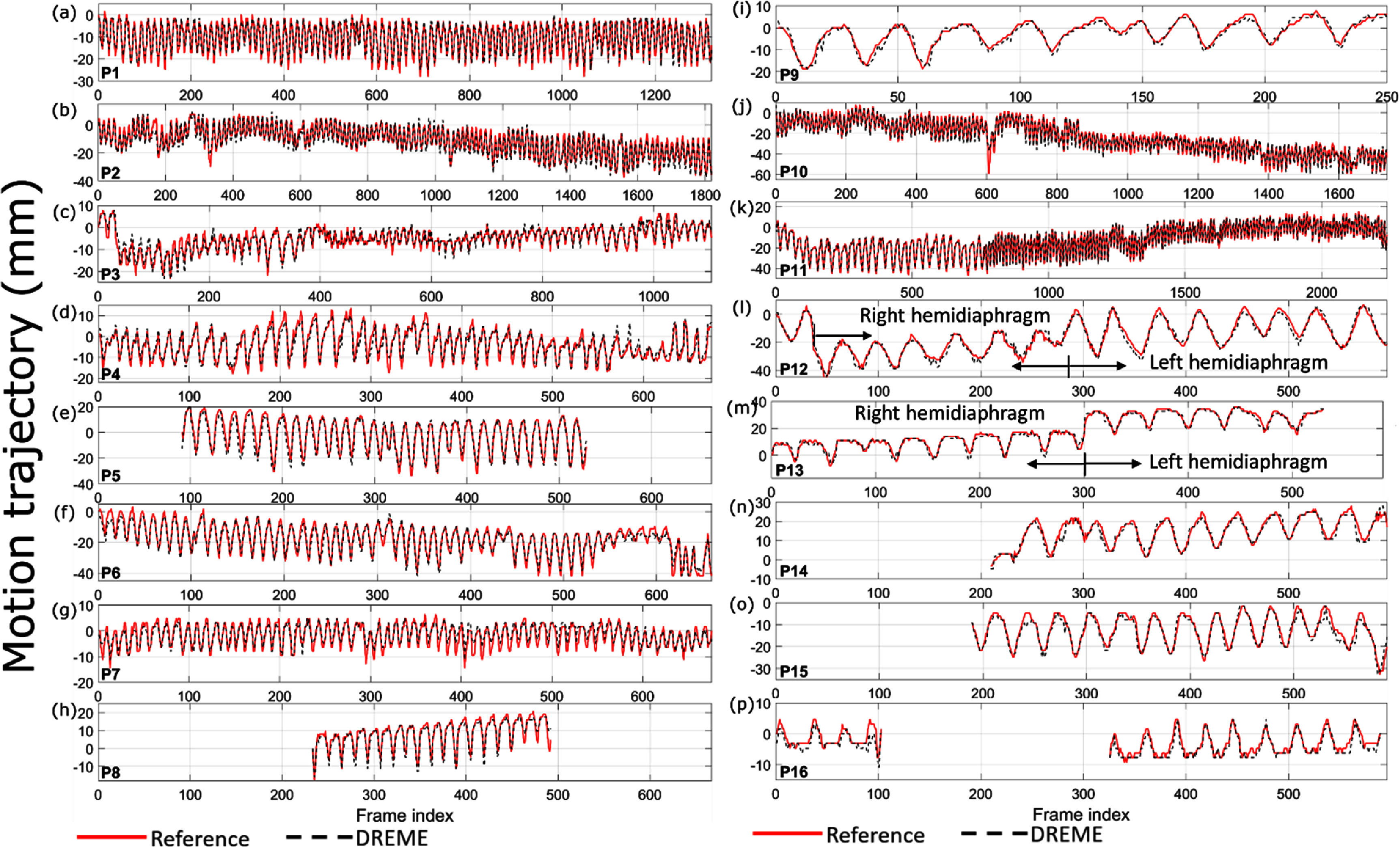
Comparison of the reference (red solid line) and DREME estimated (black dashed line) motion trajectories extracted using the AS method for the patient study. For some patients, the tracking targets may move out of the field of view, thus only partial trajectories were extracted from their AS images.

**Figure 12.**
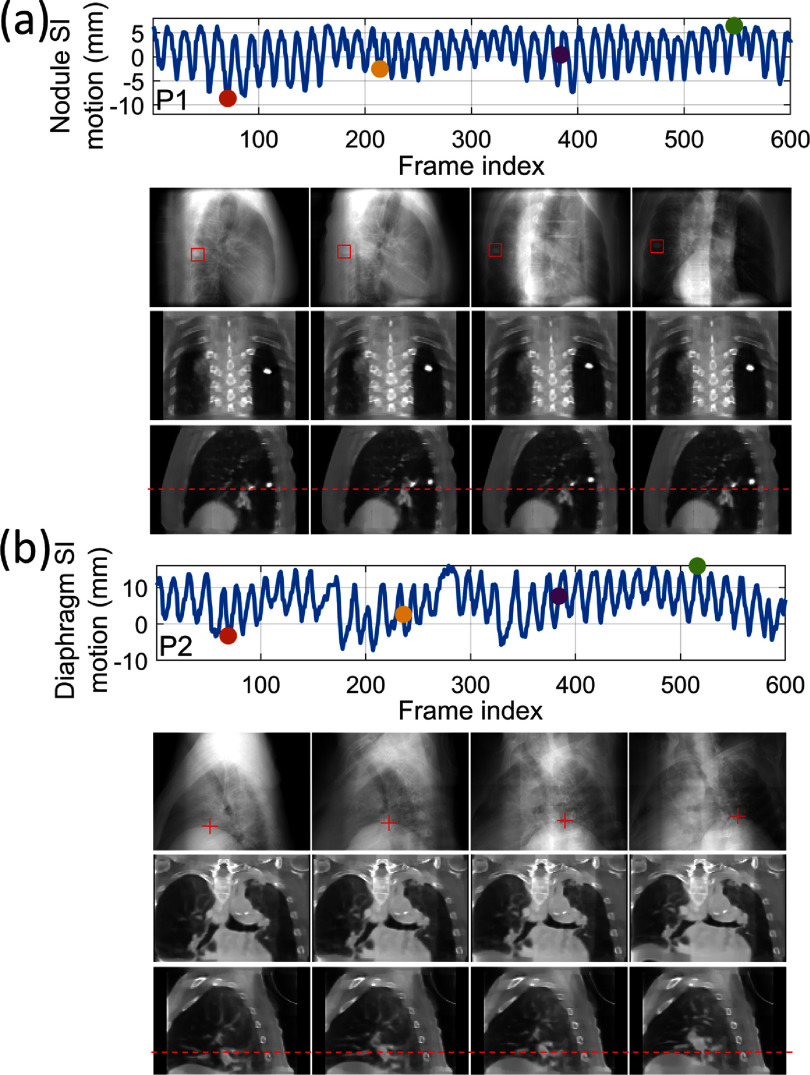
Real-time CBCTs and motion trajectories estimated by DREME for (a) P1 and (b) P2 of the patient study. The first row shows the SI motion trajectory of the tracking landmark (P1: lung nodule; P2: diaphragm apex), with the dots indicating the acquisition time points selected for plotting. The second row presents the onboard x-ray projections at the selected time points, and the ‘□’ and ‘+’ symbols indicate the 2D-projected landmark positions solved by DREME. In the following rows, real-time CBCTs at the selected time points are presented in the coronal and sagittal views.

Figure 10 presents the reconstructed reference CBCTs from the patient study. The results demonstrate that, using the ‘one-shot’ learning technique, DREME effectively reconstructs patient anatomy with diverse variations in body shapes and sizes, scan sites, and scan modes (table 2), showcasing its robustness and adaptability across various clinical settings and imaging protocols. Shading artifacts are visible in several patients (e.g. P2 and P3), mainly attributed to scatter and beam hardening effects, and the use of bow-tie filters, as no pre-processing steps were included in DREME to correct these degrading signals. The localization errors and Pearson correlation coefficients of all patients are summarized in table 7, and the motion trajectories extracted from the AS image of each patient are presented in figure 11. As shown in figure 11, DREME can accurately track various respiratory motion patterns. For all patients, the mean localization error is below 2.6 mm in the projection domain, and the estimated motion traces show high correlations (>0.89) with the reference traces. We note that the errors were evaluated in the projection domain, and the corresponding errors in the image domain would be scaled down by a factor of ∼1.5 to achieve sub-voxel localization accuracy. Figure 12 presents two examples (P1 and P2) of solved real-time CBCTs in the coronal and sagittal views.

**Table 7.** Mean localization accuracy and Pearson correlation coefficient for the SI motion trajectories extracted from the AS images for the patient study. The results are presented in terms of the mean and standard deviation (Mean ± SD) averaged over all testing projections for each patient.

Patient ID	Pearson correlation coefficient (SI trajectory)	Localization error (mm)
P1	0.946	1.5 ± 1.4
P2	0.967	2.1 ± 1.9
P3	0.919	1.4 ± 1.4
P4	0.949	1.7 ± 1.4
P5	0.964	2.6 ± 2.5
P6	0.945	1.9 ± 1.7
P7	0.887	1.5 ± 1.5
P8	0.955	1.8 ± 1.8
P9	0.965	1.2 ± 1.1
P10	0.984	2.1 ± 1.9
P11	0.993	1.2 ± 1.0
P12	0.979	2.0 ± 1.5
P13	0.986	1.3 ± 1.4
P14	0.960	1.4 ± 1.6
P15	0.962	1.4 ± 1.5
P16	0.914	1.2 ± 1.3

## 4. Discussion

In this work, we proposed a framework called DREME for real-time CBCT imaging and motion estimation in radiotherapy. The framework adopts a dual-task learning strategy (figures 1, 3, and 4) that incorporates a CNN-based real-time imaging method into a dynamic CBCT reconstruction workflow. The dynamic CBCT reconstruction solves a dynamic sequence of CBCTs from a standard pre-treatment scan to extract the latest patient anatomy and motion model, which is subsequently used for real-time CBCT motion estimation. The reconstruction algorithm is based on a joint deformable registration and reconstruction approach, and derives the motion model in a data-driven manner without relying on motion surrogate signals or patient-specific prior knowledge, eliminating the potential biases and uncertainties from these sources. To achieve a high-quality and realistic motion model, specially designed regularization terms (section 2.1.3) and a progressive multi-resolution training strategy (section 2.3, table 1) were developed. With the lightweight CNN-based motion encoder of DREME, its inference time for estimating a 3D DVF was approximately 1.5 ms, fulfilling the temporal constraint of real-time imaging (Keall *et al* 2021).

DREME was evaluated by an XCAT simulation study and a multi-institutional dataset of 16 lung patients. The XCAT results showed that DREME can capture various types of motion scenarios differing from the training scenario (figure 7), and achieved a mean tumor COME of 1.2 ± 0.9 mm (table 5). For the patient study, DREME achieved a mean localization error of 1.6 ± 1.6 mm with high Pearson correlation coefficients (table 7). The comparison study demonstrated that DREME is superior to the conventional 2D3D registration-based methods (tables 3–6 and figure 8), and also illustrated the challenges of deriving high-quality motion models from standard pre-treatment scans by conventional 4D-CBCTs. The ablation study showed that DREME’s regularization terms enhanced the quality of the motion model (tables 3–6).

Contrary to other DL-based approaches for real-time imaging, DREME does not require a large dataset or a high-quality prior motion model for training and data augmentation. Instead, DREME solves dynamic CBCTs and derives data-driven motion models in a ‘one-shot’ fashion from pre-treatment cone-beam scans. Consequently, DREME does not suffer from generalizability and stability issues, and can be utilized under various CBCT acquisition protocols, as demonstrated in the patient studies (figures 10, 11, and table 7). Unlike 4D-CT/CBCT, which over-samples anatomical information to achieve artifact-free 4D reconstructions, DREME solves the motion model from standard 3D scans, avoiding additional radiation exposure to patients. The patient study demonstrated that DREME can extract an accurate motion model from sparsely-sampled projection sets, which can further reduce patient radiation dose.

In contrast to the Type I reconstruction approach that directly reconstructs 3D volumes for real-time target localization, DREME adopts a joint deformable registration and reconstruction approach to solve the dynamic motion field for target localization. The Type I approach requires additional contouring/segmentation steps on each image volume to identify tracking targets, making its localization accuracy susceptible to target segmentation uncertainty (e.g. blurry tumor boundary). Contrarily, the DREME framework only needs to identify the target in the reference CBCT, and real-time target localization is achieved through DVF-driven contour propagation. With DREME’s deformation-driven approach, the tracking accuracy is less affected by the segmentation uncertainty associated with segmenting each real-time CBCT volume. In addition, the residual uncertainty of DREME, which is associated with reference CBCT segmentation, can be further mitigated by utilizing prior knowledge from treatment plans or complementary planning imaging modalities.

Unlike template-matching-based real-time tracking algorithms (Hindley *et al* 2019, Hirai *et al* 2020) whose localization accuracy can be sensitive to the visibility of tracking targets in projections (e.g. Shao *et al* 2022), another advantage of the DREME framework design is that the dynamic motion field estimation is based on the entire cone-beam projection. Therefore, even when the tracking target is obscured by other anatomic structures and difficult to identify in projections, the CNN-based motion encoder can still reliably infer motion by leveraging the motion correlations between visible and obscured anatomies. This capability is built via the second learning task, in which the DL-based motion encoder learns to extract meaningful motion-related image features from the whole projection in a data-driven manner. Figure 12(a) demonstrates that even though the tracking target (i.e. lung nodule) was obscured by the spine in the lateral-direction projections (i.e. the first two panels of the second row), DREME could still accurately estimate its motion (cf figure 11(a)). In addition, we did not observe a significant projection angle dependence of the tumor COME in the XCAT study (section II in the supplementary materials), where we found the view obstruction from the spine for the lateral-direction projections did not affect the tumor tracking accuracy.

DREME is designed to be trained on a pre-treatment CBCT scan and tested on intra-treatment x-ray projections, with the imaging data all coming from the same treatment session. However, such a scheme is challenging to evaluate, due to the lack of repeated CBCT scans within the same treatment session, as the second scan will incur additional radiation dose to the patient. Therefore, we divided the pre-treatment CBCT scan of each patient into non-overlapping training and testing projection sets, to simulate two scans acquired in the same treatment session. To evaluate DREME on more-independent scans, we tested the robustness and performance of DREME on two CBCT scans acquired during different treatment sessions for three patients (P4–P6) from the SPARE dataset (section III of supplementary materials). These patients had multiple independent CBCT scans, which were acquired on different treatment days. Thus, the training/testing CBCT projection sets may not harbor the same anatomy/motion model (as assumed by DREME) but present a scenario of independent scans close to the desired evaluation setting. The results showed that the localization error slightly increased, compared to the localization error tested on the pre-treatment scans (table 7). Such a degradation in accuracy is expected, as the training and testing projection sets do not belong to the same treatment session, and may contain different anatomy/motion models. The DREME is expected to perform better when the two independent CBCT projection sets come from the same treatment session.

To address the inherent challenges of the ill-posed spatiotemporal inverse problem and to facilitate effective learning, we employed a low-rank, B-spline-based motion model to regularize the reconstruction problem. However, our motion model has potential limitations in its application. First, the low-rank representation (equation (2)) assumes that anatomic motion fields can be described by a linear superposition of a small number of MBCs ${{\boldsymbol{e}}_i}\left( {\boldsymbol{x}} \right)$. Rare motion events besides respiration, such as sudden coughing, swallowing, or intestine peristalsis, may not be fully captured by such a representation. As our study focuses on the thoracic-abdominal region, we used three MBCs to describe the dominant respiratory motion, which was demonstrated sufficient (Li *et al* 2011). For other motion types (e.g. heart beating), more MBCs or a more flexible motion model may be needed for accurate motion characterization. Second, the MBCs were represented by a B-spline interpolant, which assumes that the spatial distributions of the MBCs are smooth and continuous functions of the voxel coordinate ***x***. Therefore, it can be challenging to accurately describe the discontinuous sliding motion of organs against surrounding body walls (Loring *et al* 2005, Al-Mayah *et al* 2009). We used a 24 × 24 × 24 grid of control points for the highest MBC level to describe motion with a high spatial resolution, which yielded satisfying accuracy. While it is possible to further increase the number of control points to describe more complex and potentially discontinuous motion, it may increase the chance of overfitting and remains to be investigated.

Our joint image reconstruction and registration approach also assumes no time-dependent image contrast variations during the pre-treatment CBCT scan and the following radiation treatment. Consequently, the current DREME framework is not suitable for contrast-enhanced dynamic CBCT reconstruction or real-time imaging, where tissue contrast is dynamically affected by the perfusion and clearance of the contrast agent.

As DREME relies on data-driven motion models for real-time motion estimation, the quality of the motion model directly influences the accuracy of the motion estimation. We observed a correlation between the quality of solved pre-treatment dynamic CBCTs and the performance of real-time motion estimation. Therefore, a direction for future work is to further improve the quality and robustness of the reconstructed CBCTs and the data-driven motion models. A major limitation of the DREME framework is the time for model training. The training time of DREME (based on CBCTs of 200 × 200 × 100 voxels) was approximately four hours on an Nvidia V100 GPU card. Several approaches could potentially be employed to accelerate the training process. Since the DREME network was trained from scratch for each projection set, transfer learning could reduce the training time. For example, the CNN-based motion encoder could be replaced by a pre-trained network (e.g. ResNet), and then fine-tuned for each case. Alternatively, one could pre-train the motion encoder using the cone-beam scan from a previous fraction/treatment, then fine-tune the motion encoder using the current pre-treatment scan of the same patient, provided no dramatic variations in patient anatomy and motion. In addition, the cone-beam projector from the ASTRO toolbox parallelizes DRR simulations across multiple gantry angles, whereas DREME only requires a single DRR at each gantry angle. Consequently, DREME sequentially simulated DRRs for the dynamic sequence to calculate the similarity loss. A more efficient parallelization scheme for the cone-beam projector tailored to the DREME framework could also speed up the model training. Furthermore, a more efficient approach for evaluating projection-domain similarity loss could also be employed. Currently, all DRR pixels are computed to quantify the similarity between the DRRs and x-ray projections. However, pixels do not play an equal role in the similarity quantification. For instance, the upper thorax typically exhibits smaller amplitudes and simpler patterns of respiratory motion than the lower thorax and the diaphragm region. Therefore, a better sampling scheme could be designed to increase the sampling rate of pixels exhibiting large and complicated motion. Since the above approaches require major modifications of the algorithms in the current workflow and are beyond the scope of the study, we leave these possibilities for future investigations.

## 5. Conclusion

We proposed a joint framework for real-time CBCT imaging and motion estimation. The proposed method reconstructed a dynamic sequence of CBCTs from a pre-treatment scan, and subsequently used the latest anatomy and motion model for real-time motion estimation to avoid potential biases associated with patient-specific prior knowledge. The results demonstrated that the framework can yield high-quality motion models and achieve sub-voxel localization accuracy in both simulation and real patient studies. The framework can have broad applications in radiotherapy, as the derived real-time motion allows potential real-time tumor tracking, dose accumulation, and adaptive radiotherapy.

## Data Availability

The data cannot be made publicly available upon publication because they contain sensitive personal information. The data that support the findings of this study are available upon reasonable request from the authors.
